# Development and Validation of Prognostic Model for Lung Adenocarcinoma Patients Based on m6A Methylation Related Transcriptomics

**DOI:** 10.3389/fonc.2022.895148

**Published:** 2022-06-16

**Authors:** Huijun Li, Song-Bai Liu, Junjie Shen, Lu Bai, Xinyan Zhang, Jianping Cao, Nengjun Yi, Ke Lu, Zaixiang Tang

**Affiliations:** ^1^ Department of Biostatistics, School of Public Health, Medical College of Soochow University, Suzhou, China; ^2^ Department of Medical Biotechnology, Suzhou Key Laboratory of Medical Biotechnology, Suzhou Vocational Health College, Suzhou, China; ^3^ Jiangsu Key Laboratory of Preventive and Translational Medicine for Geriatric Diseases, Medical College of Soochow University, Suzhou, China; ^4^ School of Data Science and Analytics, Kennesaw State University, Kennesaw, GA, United States; ^5^ School of Radiation Medicine and Protection and Collaborative Innovation Center of Radiation Medicine of Jiangsu Higher Education Institutions, Soochow University, Suzhou, China; ^6^ Department of Biostatistics, University of Alabama at Birmingham, Birmingham, AL, United States; ^7^ Department of Orthopedics, Affiliated Kunshan Hospital of Jiangsu University, Suzhou, China

**Keywords:** prognostic model, lung adenocarcinoma, m^6^A, immunotherapy, drug prediction.

## Abstract

Existing studies suggest that m^6^A methylation is closely related to the prognosis of cancer. We developed three prognostic models based on m^6^A-related transcriptomics in lung adenocarcinoma patients and performed external validations. The TCGA-LUAD cohort served as the derivation cohort and six GEO data sets as external validation cohorts. The first model (mRNA model) was developed based on m^6^A-related mRNA. LASSO and stepwise regression were used to screen genes and the prognostic model was developed from multivariate Cox regression model. The second model (lncRNA model) was constructed based on m^6^A related lncRNAs. The four steps of random survival forest, LASSO, best subset selection and stepwise regression were used to screen genes and develop a Cox regression prognostic model. The third model combined the risk scores of the first two models with clinical variable. Variables were screened by stepwise regression. The mRNA model included 11 predictors. The internal validation C index was 0.736. The lncRNA model has 15 predictors. The internal validation C index was 0.707. The third model combined the risk scores of the first two models with tumor stage. The internal validation C index was 0.794. In validation sets, all C-indexes of models were about 0.6, and three models had good calibration accuracy. Freely online calculator on the web at https://lhj0520.shinyapps.io/LUAD_prediction_model/.

## Introduction

Lung cancer ranks as the major cause of cancer death, accounting for almost a quarter of cancer deaths (1). Lung adenocarcinoma (LUAD) is the most common subtype of lung cancer, accounting for more than 40% of lung cancer incidence (2).N^6^-methyladenosine (m^6^A), the most abundant form of posttranscriptional RNA modification in eukaryotes, plays an important role in a variety of biological processes by regulating the translation, processing, splicing, stabilization, and degradation of target RNA (3). The abundance and effects of m^6^A methylation modification on RNA are maintained by its methyltransferases (‘writers’), binding proteins (‘readers’), and demethylases (‘erasers’) (4).

Existing studies suggest that m^6^A methylation is closely related to the prognosis of cancer. An increasing number of m^6^A-related genes have been developed as molecular markers of cancer prognosis. In lung adenocarcinoma, several biomarkers have also been developed. Some of the biomarkers are based on single gene model, such as *YTHDC2 (* 5), *NPM1 (* 6) and *LCAT3 (* 7). Some others are multigene-based, including Wang (5 genes) (8), Sun (10 genes) (9), and Zhu (6 genes) (10). Such molecular biomarkers have been shown to enhance the accuracy of overall survival (OS) prediction in LUAD.

However, the predictive power of these markers is often limited. First, most models were constructed based only on mRNAs or lncRNAs. Second, most of the models lack some key parameters, prognostic index or baseline survival function, which make it difficult for others to validate or use them. Further on, even if complete parameters related to model validation are provided (unfortunately, none is found in prediction model related to lung adenocarcinoma at present), few convenient online interaction tools are available.

Based on the above fact, we attempted to develop models to fill in the gaps in prognostic model of lung adenocarcinoma using m^6^A-related transcriptomics to predict OS. First, we developed a mRNA prognostic model and a lncRNA prognostic model for lung adenocarcinoma on TCGA cohort and evaluated the two models on several GEO data sets. And then we used the two models and some clinical variables as alternative predictors to construct a multi-omics clinical prediction model. All prediction models developed have two to six independent external validation sets. To further facilitate the practical application of the constructed prediction model in clinical practice, we developed a free online calculator: https://lhj0520.shinyapps.io/LUAD_prediction_model/.

## Methods

### Data Acquisition and Processing

For model derivation, we downloaded RNA-seq data (counts values) of 585 LUAD patients (version: 07-20-2019) and corresponding clinical information (version: 08-07-2019) in GDC TCGA from the UCSC Xena public data hub (http://xena.ucsc.edu/). A total of 486 samples with primary tumors and overall survival greater than 30 days were retained. The expression data from the TCGA data portal were quantile normalized and log2-transformed (11). In addition, the somatic mutation data of LUAD patients were also downloaded as a mutation annotation format (MAF) file from GDC Data Portal (https://portal.gdc.cancer.gov/).

For model validation study, 6 datasets from GEO (https://www.ncbi.nlm.nih.gov/geo/) database were considered, including GSE29016 (GPL6947, n=38), GSE29013 (GPL570, n=30) GSE3141 (GPL570, n=58), GSE30219 (GPL570, n=85), GSE37745 (GPL570, n=106), and GSE50081 (GPL570, n=127). We downloaded the series matrix files and their platform annotation information. All the microarray data were quantile normalized and the Robust Multichip Average (RMA) method was used for background adjusted (12).

### Annotation of LncRNA Expression

The lncRNAs were extracted according to file downloaded from GENCODE project (https://www.gencodegenes.org/, release 37).

### Selection of m^6^A Methylation Regulators and m^6^A-Related mRNAs

We obtained m^6^A methylation regulators from the literature (13). For m^6^A-related genes in LUAD, genes annotated as ‘protein coding’ were retained from the m6AVar database (http://rmvar.renlab.org/) (14), which is a comprehensive database of m^6^A-associated variants.

### Selection of m^6^A-Related LncRNAs

Spearman rank correlation analysis was conducted between m^6^A methylation regulatory factors and lncRNAs. Rank correlation coefficient | R_s_ | >0.3 and P <0.05 were used as the selection criteria.

### Development and Validation of Model Based on mRNAs

Using the mRNA dataset of TCGA LUAD patients as a derivation cohort, we developed a prognostic model to predict OS. As the first step of variable selection, the least absolute shrinkage and selection operator (LASSO) method (15) of R package ‘glmnet’ was used to reduce the dimension of genes. The optimal value of λ was selected by tenfold cross-validation, and corresponding variables with nonzero coefficients were retained. Next, the “stepAIC” function with “both” in the R package “MASS” was applied to perform stepwise Cox regression (16) for the retained genes, and the optimal gene combination was obtained according to the lowest Akaike information criterion (AIC) value.

Based on the obtained Cox model, the risk score, i.e., prognostic index (PI), could be calculated directly using the “predict” function in R package “rms” with the parameter “type= lp” (17). The calculation formula is as follows:


(1) $$Risk\;Score\;\left(PI\right)=\left(\sum_{i=1}^{n}\beta_{i}*Exp_{i}\right)-\bar{x},$$

where n refers to the total number of genes in the model; *β_i_* refers to the coefficient of each gene; and *Exp_i_* refers to the expression level of each gene; $\bar{x}$ refers to the mean of PI.

There are two fundamental aspects, discrimination and calibration, to evaluate the performance of the model. Discrimination refers to the ability of a model to differentiate between high-risk patients and low-risk patients (18). It is represented by Harrell’s c-index of concordance (C-index) (19). Internal validation adopted bootstrapping (1000 resamples). The C-index was calculated by the “validate” function in the R package “rms” (17). Time-dependent ROC curves at 1-, 3- and 5-year were created by the “survivalROC” R package (20). Through the “cindex” function of the “pec” R package (21), the dynamic time-dependent C-index curve of each dataset was plotted. Calibration refers to the agreement between the predicted and observed survival probabilities (18). The calibration plot was applied to assess the calibration of our model at 1, 3 and 5 years respectively by the “rms” R package (17).

In addition, we estimated the baseline survival function, S_0_(t) which is an essential indicator for prediction model (22) and presented it by Kaplan–Meier curves. For the Cox proportional hazards model, the survival probability at different time points are calculated by the following formula (23):


(2) $$S\left(t|X\right)=S_{0}{\left(t\right)}^{exp\left(PI\right)},$$

where S(t|X) denotes the predicted survival at time t for a patient with predictors X; S_0_(t) denotes the baseline survival function; and PI denotes the linear predictors. The baseline survival is estimated as , *S*_0_(*t*) = *exp*[-H_0_(t)] where H_0_(t) is the baseline cumulative hazard (22). It can be computed by the “basehaz” function in the “survival” R package (24).

The baseline survival function is crucial, which loads the information needed to evaluate the calibration of survival probabilities in the derivation dataset and more than that calibration in validation datasets (22). Therefore, if we want to validate the Cox model, it is necessary to know the baseline survival function and regression coefficient of the model.

The “surv_cutpoint” function in the R package “survminer” was used to determine the appropriate cutoff value of PI based on the maximum rank statistics (25), and patients in each data set were divided into two risk groups. The predicted survival curve of each person could be calculated by the baseline survival probability. Then, the calibration accuracy of the model can also be evaluated by comparing the average predicted survival probability curve with the observed survival probability curve in the two risk groups (18).

The mRNA model has four GEO external validation sets. Three single data sets included: GSE37745 (n=106), GSE29016 (n=38), and GSE50081 (n=127). Another dataset was pooled by five datasets (GSE3141, GSE29013, GSE30219, GSE37745 and GSE50081). The combined dataset was adjusted for batch effect through the “ComBat” function of the “sva” R package (26). We referred to this combined dataset as the “GSE5total” dataset.

### Development and Validation of Model Based on LncRNAs

For lncRNA model, we used four steps to obtain appropriate lncRNAs. First, the random survival forest (RSF) (27), a machine learning method for regression, was used to conduct preliminary feature screening for m^6^A-related lncRNAs through “rfsrc” function of “randomForestSRC” R package (28). This algorithm was used to rank prognostic lncRNAs (ntree =1000), and we selected the top 100 lncRNAs for the next step of selection. Second, we applied LASSO to shrink variables. Then, the prognostic factors retained by the LASSO algorithm were analyzed by best subset selection. To realize this method in the Cox proportional hazards model, we used the R package “BeSS” (29). Finally, stepwise Cox regression was used to select the optimal model from the factors obtained in the previous step.

The performance evaluation and PI calculation methods of lncRNA model were the same as mRNA model.

Two datasets, GSE30219 (n=85) and GSE50081 (n=127), were used to validate the lncRNA model. For expanding the sample size of the validation set, we combined the above two data sets into one data set and named it “GSE2total” to validate.

### Development and Validation of Comprehensive Prediction Model

To further expand the clinical prediction capacity of m^6^A-related model, we decided to develop a more comprehensive clinical prediction model (we called it the “comprehensive prediction model”) by combining two risk scores obtained from the above models with clinical variables.

We used multiple imputation by chained equations of the R package “mice” to impute the missing values of clinical variables (5 times) (30). The number of iterations in each imputation was five by default. The variables used in the multiple imputation model included the two risk scores(mRNA risk score and lncRNA risk score), three clinical factors that were common in the derivation and validation datasets (age, sex and tumor stage) and the outcome (the Nelson–Aalen estimator of the baseline cumulative hazard and the outcome indicator) (31, 32). For 5 imputed data sets, we put each imputed set below each other into a stacked data with a weight of 1/5 per patient (5 means number of imputation) (33).

The predictive factors in the multivariate Cox regression model were screened by stepwise regression. The performance evaluation and PI calculation methods of this model were still the same as mRNA model. Two datasets from GEO database, GSE37745 (n=106) and GSE50081 (n=127), were used to validate.

### Somatic Mutation Analysis

The “maftools “R package was used to analyze TCGA somatic mutation data (34).

### Immunotherapy Exploration of the Model

Immune checkpoints, negative regulators of immune activation, can downregulate the immune state of the body and limit antitumor responses (35, 36). Tumor Immune Dysfunction and Rejection (TIDE) is a computational framework developed to assess the potential of tumor immune escape from gene-expressed cancer samples and to measure the responsiveness of immune checkpoint inhibitors (37, 38). TIDE scores were calculated for each of 486 LUAD patients by the TIDE website (http://tide.dfci.harvard.edu/).

### Drug Prediction

By using the “calcPhenotype” function of the R package “oncoPredict” (39) and the database resources of Genomics of Drug Sensitivity in Cancer (GDSC) V2 as development data, six commonly used chemotherapy drugs (paclitaxel, fluorouracil, cisplatin, vinorelbine, gemcitabine, and docetaxel) were used for analysis, and the half-maximum inhibitory concentration (IC50) of each drug was estimated in every sample.

### Statistical Analysis

All statistical analyses were performed using R (version 4.1.0). A bivariate normal distribution test was performed on the data requiring correlation analysis. The Shapiro-Wilk test and Bartlett’s test of homogeneity of variances were performed on the data requiring comparison between groups. Student’s t test was used if the continuous variable was normally distributed, and the Wilcoxon rank sum test was used if the continuous variable was not normally distributed. P < 0.05 was considered statistically significant. Median follow-up time was calculated by reverse Kaplan-Meier method (40). The survival curves were analyzed using log-rank test.

## Results

### Patient Cohorts

**Figure 1:**
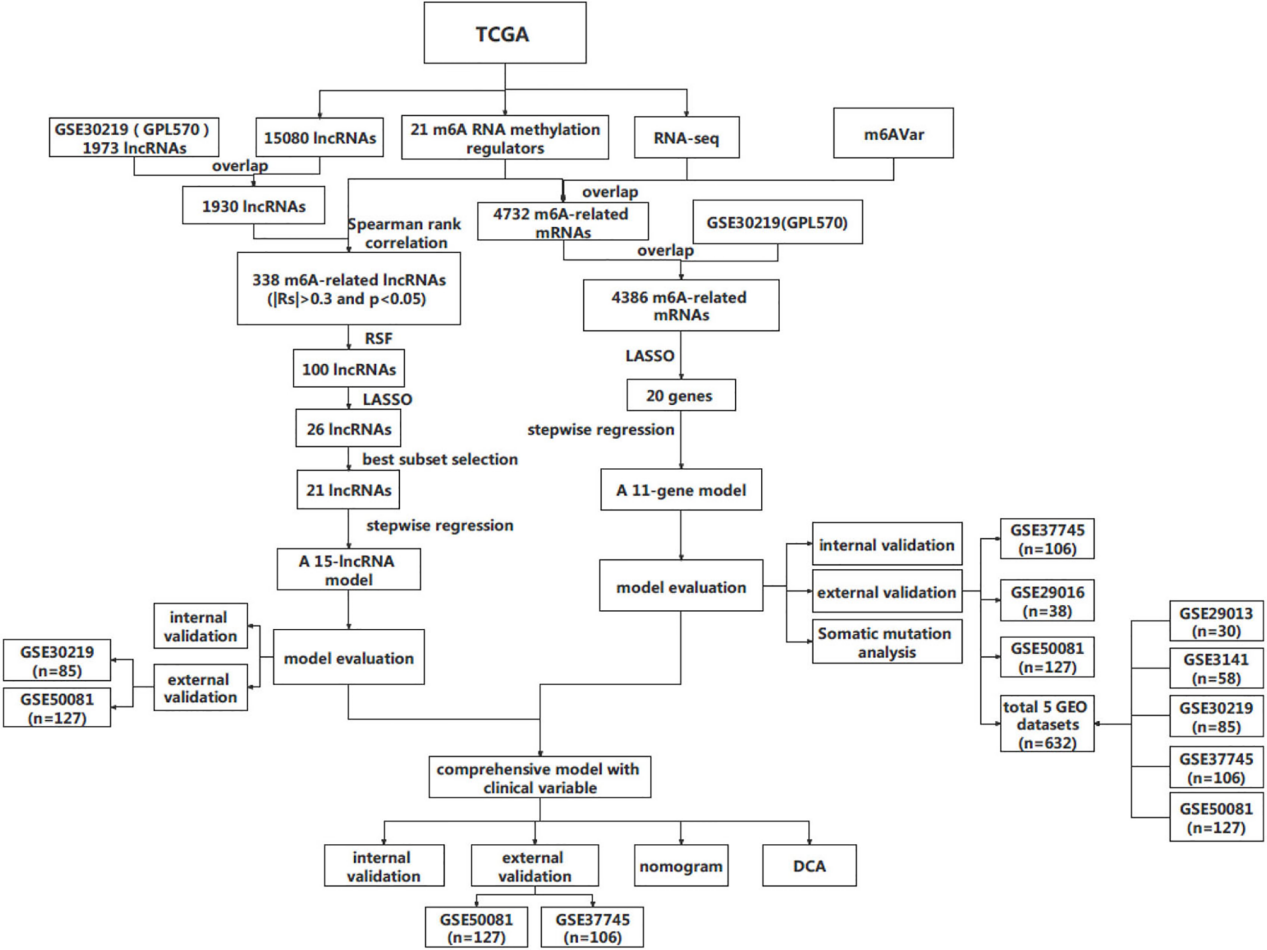
The workflow of this study. RSF, random survival forest; DCA, decision curve analysis.

The design and workflow of the models constructed in this study are shown in **Figure 1**. The patient characteristics are summarized in **Table 1**. For the derivation cohort, a total of 486 patients had 175 deaths and an event rate of 36%, with a median overall survival of 2.4 years (95%CI: 2.2-2.8).

**Table 1:** Patient characteristics.

Characteristic	Derivation Cohort	Validation Cohorts					
	TCGA (n=486)	GSE29016(n=38)	GSE30219(n=85)	GSE37745(n=106)	GSE50081(n=127)	GSE5 total(n=406)	GSE2 total(n=212)
Age, year (IQR)	66.0 (59.0,72.0)	69.0 (59.0,73.0)	60.0 (55.0,69.0)	64.0 (55.0,70.0)	69.9 (62.8,75.7)	–	–
Missing values, n (%)	10 (2.0)	–	–	–	–	–	–
Gender (%)							
Female	261 (54)	20 (53)	19 (22.4)	60 (56.6)	62 (48.8)	–	–
Male	225 (46)	18 (47)	66 (77.6)	46 (43.4)	65 (51.2)	–	–
Tumor stage (%)							
Stage I	261 (53.7)	29 (76)	–	70 (66.0)	92 (72.4)	–	–
Stage II	114 (23.4)	6 (16)	–	19 (17.9)	35 (27.6)	–	–
Stage III	79 (16.2)	2 (5.3)	–	13 (12.3)	–	–	–
Stage IV	25 (5.1)	–	–	4 (3.8)	–	–	–
Missing values, n (%)	7 (0.01)	1 (2.6)	–	–	–	–	–
Follow-up time, years (95%CI)	2.4 (2.2, 2.8)	11.8 (11.4, 13.4)	9.7 (8.3,11.2)	10.5 (9.2,13.0)	5.5 (5.2,6.0)	6.2 (5.8, 6.8)	6.2 (5.8,6.8)
Death events (%)	175 (36)	28 (73.7)	45 (52.9)	77 (72.6)	51 (40.1)	213 (52.5)	96 (45.3)

In the comprehensive prediction model, the number of events per variable in derivation model was 35 (175/5), indicating a reasonable number of events compared to the number of candidate predictors. This quantity meets the EPV principle required by the sample size of the prediction model, that is, there should be at least ten events per variable (23). We observed only a slight percentage of missing values for age and tumor stage in the TCGA cohort, 2.1% and 1.4%, respectively (**Figure S1A**). **Figure S1B** shows that the missing values of the data variables correspond to random missing values (41). All 486 patients who met the requirements for the development data were included in the model after imputation.

**Figure 2:**
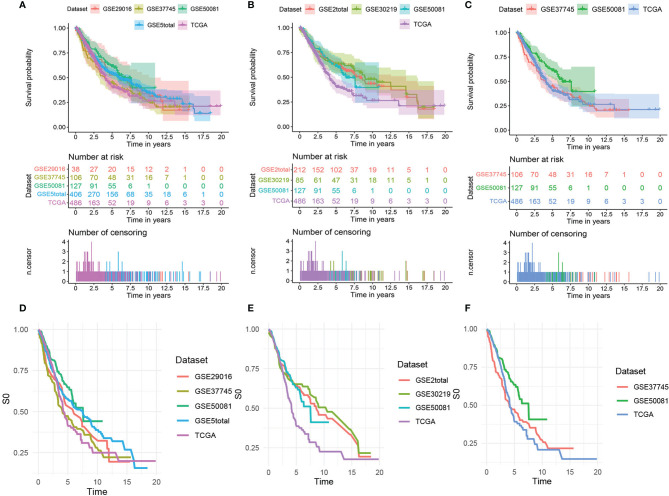
The survival curves and baseline survival probability curves of each data set in the three models. The survival curves of each data set in **(A)** the mRNA model, **(B)** the lncRNA model, and **(C)** the comprehensive clinical model. The baseline survival probability curves of each data set in **(D)** the mRNA model, **(E)** the lncRNA model, and **(F)** the comprehensive clinical model.

**Figure 2** shows the survival curves (**Figures 2A–C**) and baseline survival probability curves (**Figures 2D–F**) of each data set in the three models.

### Development and Validation of the mRNA Model

**Figure 3:**
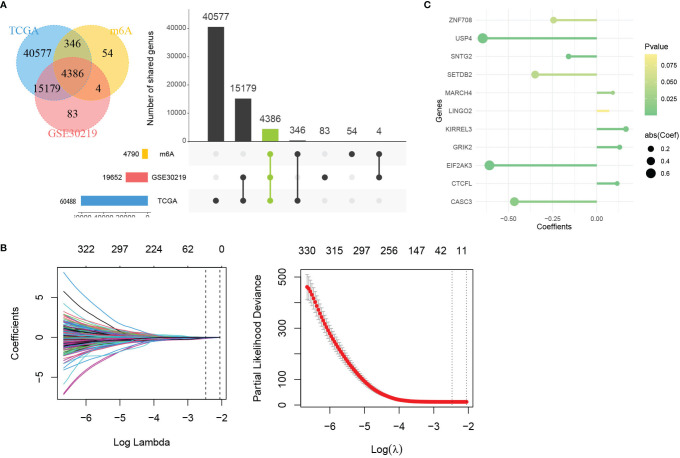
Identification of genes in mRNA model. **(A)** Venn plot of 4386 mRNAs related to m^6^A. **(B)** LASSO shrinking path diagram. **(C)** The coefficients of 11 genes in the model.

The 21 m^6^A regulatory factors extracted from the literature are listed in **Table S1**. Common genes obtained from the three data sets m6AVar, TCGA and GSE30219 and 21 regulatory factors were included; finally, we obtained 4386 mRNAs related to m^6^A (**Figure 3A**).

These genes were screened by LASSO (**Figure 3B**) and stepwise regression successively, and a prediction model containing 11 mRNAs associated with OS was obtained (CASC3, USP4, CTCFL, SETDB2, MARCH4, KIRREL3, GRIK2, EIF2AK3, SNTG2, LINGO2 and ZNF708). **Figure 3C** shows the coefficients of the model visually. Based on the genes and coefficients in the development data set, PI was constructed as follows:


$$\begin{array}{ccccc} PI=-0.46605\times CASC3-0.64556\times USP4+0.11549\times CTCFL & \;-0.34872\times SETDB2+0.09105\times MARCH4 & +0.16502\times KIRREL3+0.12956\times GRIK2-0.60740\times EIF2AK3 & \;-0.15933\times SNTG2+0.06450\times LINGO2-0.24452\times ZNF708 & \;+23.20828 \end{array}$$

The distribution of PI in the derivation and validation data sets were shown in **Figure S2A**. The base survival probability of the mRNA model from 1 to 10 years was given in **Table S2**. By substituting the calculated PI and the basic survival probability at different time points into formula (2), the prognostic survival probability of individual at corresponding time points can be obtained

**Figure 4:**
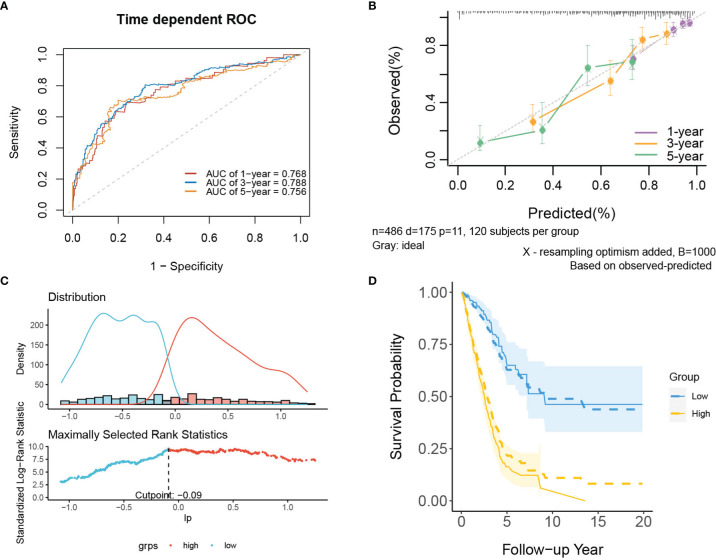
The performance of the mRNA model in the derivation dataset. **(A)** 1-,3-,5-year ROC curves and **(B)** calibration plot of the mRNA model. **(C)** The optimal cutoff value of PI. **(D)** Predicted versus observed survival probability in per risk group. Solid line: observed Kaplan-Meier curve; dotted line: average predicted survival curve; shaded area: 95% confidence interval of observed survival probability.

In internal validation, the apparent C-index of the model was 0.751(95%CI:0.711-0.791), and the optimism-corrected C-index with 1000 bootstrap resamples was 0.736. The 1-year, 3-year and 5-year AUCs of the model were 0.768, 0.788, and 0.756, respectively (**Figure 4A**). The calibration plot shows that the model has good agreement between predicted and observed survival probabilities at 1, 3 and 5 years (**Figure 4B**). In addition, patients were divided into two risk groups based on the optimal cutoff value of PI (**Figure 4C**). In **Figure 4D**, the observed Kaplan–Meier survival curves (the solid line) were close to the average predicted survival curves (the dotted line) in the two risk groups, which also proved that our prediction model had good calibration accuracy. **Figure S3** shows the Kaplan–Meier survival curves (**Figure S3A**) and risk factor association diagrams (**Figure S3B**) for the two risk groups.

**Figure 5:**
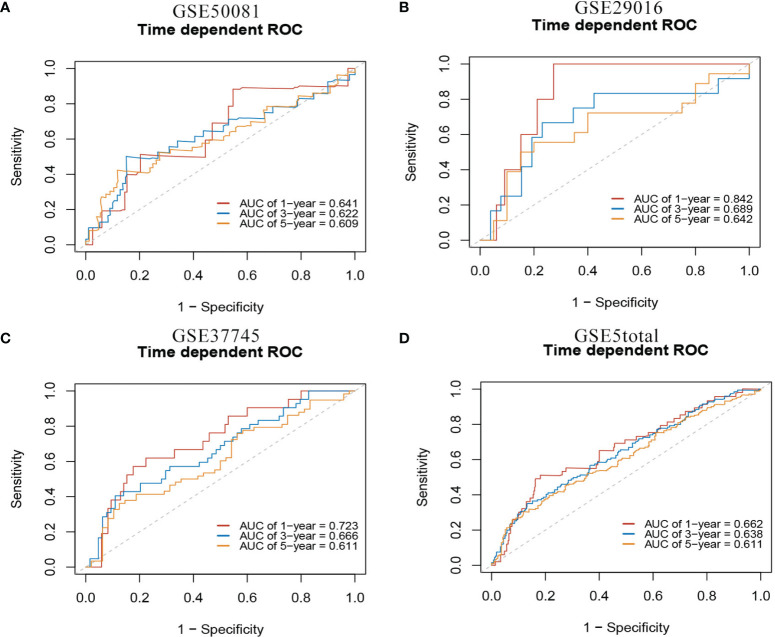
1-,3-,5-year ROC curves of mRNA model in external validation data sets. **(A)** GSE50081 dataset. **(B)** GSE29016 dataset. **(C)** GSE37745 dataset. **(D)** GSE5total dataset.

**Figure 6:**
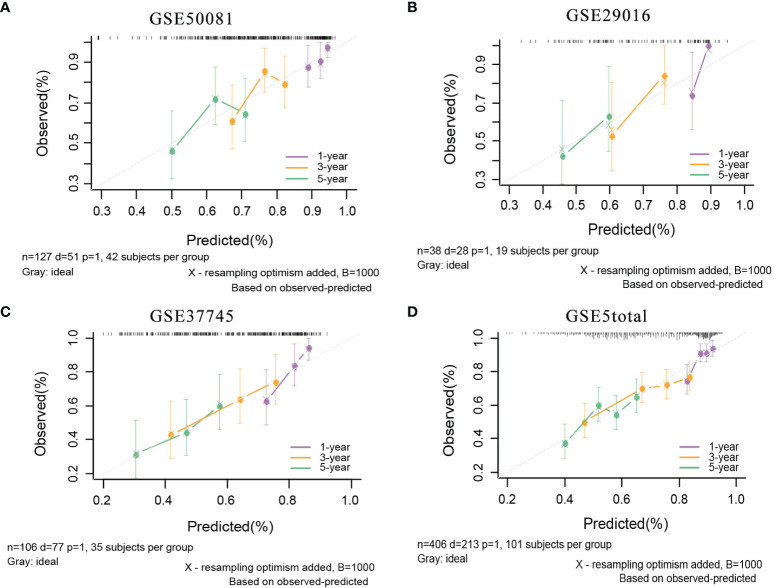
The calibration plots of mRNA model in external validation data sets. **(A)** GSE50081 dataset. **(B)** GSE29016 dataset. **(C)** GSE37745 dataset. **(D)** GSE5total dataset.

In the external validation cohorts, C indexes of the model were acceptable, which were 0.598(95%CI:0.511-0.685) (GSE50081), 0.608(95%CI:0.510-0.707)(GSE29016), 0.634(95%CI:0.571-0.697)(GSE37745) and 0.608(95%CI:0.567-0.649)(GSE5total). In addition, **Figure S4A** shows C-indexes of the model over 1-10 years in all datasets. According to the time-dependent ROC curves (**Figure 5**), the area under the curves of the model in the four validation sets of 1, 3 and 5 years were all above 0.6, which also indicated that its discriminative ability is satisfactory. The calibration diagrams from the four validation sets show the good calibration accuracy of the model in external validation (**Figure 6**). Patients in the validation sets were divided into two risk groups based on the maximum rank statistics (**Figures S5A–D**), and the average predicted survival curves (the dotted line) and observed survival curves (the solid line) of the two groups were compared to further verify the calibration accuracy of the model (**Figures S5E–H**). The long-term prediction ability of the model in the GSE50081 (**Figure S5E**) and GSE29016 (**Figure S5F**) datasets was not as good as that in the other two datasets (**Figures S5G–H**). However, within 5 years, the calibration accuracy of the model is acceptable. Subsequently, the Kaplan–Meier survival curves of the two risk groups and the risk factor association diagrams of the model in each validation set are shown in **Figures S6, S7** respectively.

### Development and Validation of the lncRNA Model

**Figure 7:**
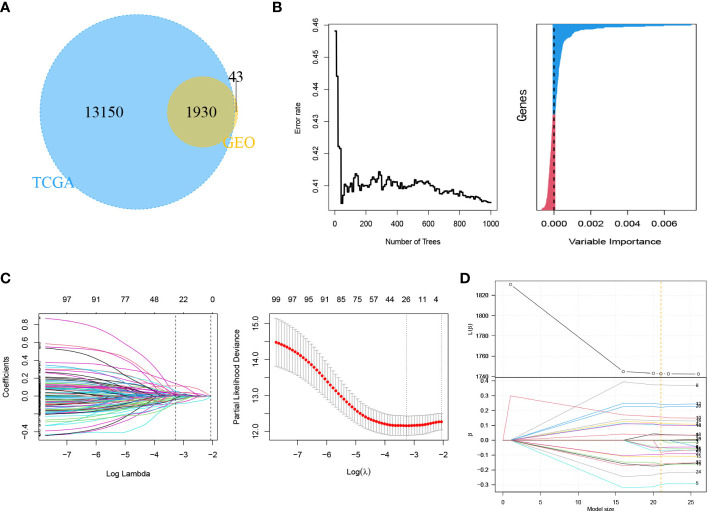
Identification of genes in lncRNA model. **(A)** Venn plot of 1930 lncRNAs related to m^6^A. **(B)** Random survival forest analysis. **(C)** LASSO shrinking path diagram. **(D)** The coefficient profile plot of the coefficient and loss paths for best subset selection.

**Figure 8:**
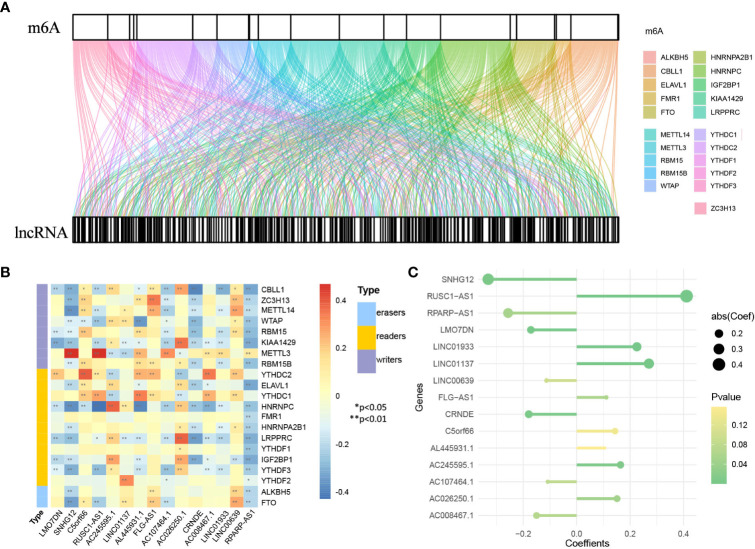
Identification of genes in lncRNA model. **(A)** Sankey diagram of 21 m^6^A regulators and 1930 m^6^A-related lncRNAs. **(B)** The heatmap for the correlation between 21 m^6^A genes and 15 prognostic m^6^A-related lncRNAs. **(C)** The coefficients of 15 lncRNAs in the model.

First, 1930 common lncRNAs of TCGA and GSE30219 data sets were obtained (**Figure 7A**). Then, genes were screened by the importance score of random survival forest (**Figure 7B**), and the top 100 genes were reserved for the next step. Twenty-six genes were obtained by LASSO screening of 100 reserved genes (**Figure 7C**). Next, we selected the best subset selection method for further screening of genes and obtained 21 genes (**Figure 7D**). Finally, 15 lncRNAs of the prediction model associated with OS were obtained by stepwise regression (*SNHG12, RPARP-AS1, CRNDE, LMO7DN, AC008467.1, LINC00639, AC107464.1, AL445931.1, FLG-AS1, C5orf66, AC026250.1, AC245595.1, LINC01933, LINC01137, RUSC1-AS1*). Furthermore, the co-expression networks of 21 m^6^A and 1930 lncRNAs were visualized by a Sankey diagram, as shown in **Figure 8A**. In addition, the heatmap of the correlation between 21 m^6^A genes and 15 lncRNAs in the model is shown in **Figure 8B**.

Based on the genes and coefficients in the development data set, PI was constructed as follows:


$$\begin{array}{lllllll} PI=-0.17135\times LM07DN-0.33117\times SNHG12+0.14349\times C5orf66 & \;+0.41125\times \left(RUSC1-AS1\right)+0.16394\times AC245595.1 & \;+0.27029\times LINC01137+0.10490\times AL445931.1 & \;+0.11064\times \left(FLG-AS1\right)-0.10828\times AC107464.1 & \;+0.15101\times AC026250.1-0.17919\times CRNDE & \;-0.15018\times AC008467.1+0.22517\times LINCO1933 & \;-0.11297\times LINC00639-0.25657\times \left(PRARP\;-\;AS1\right)-\;0.07307 \end{array}$$

**Figure 8C** shows the coefficients of the model visually. The distribution of PI in the development data set and validation set is shown in **Figure S2B**. The base survival probability of the lncRNA model from 1 to 10 years is given in **Table S2**

**Figure 9:**
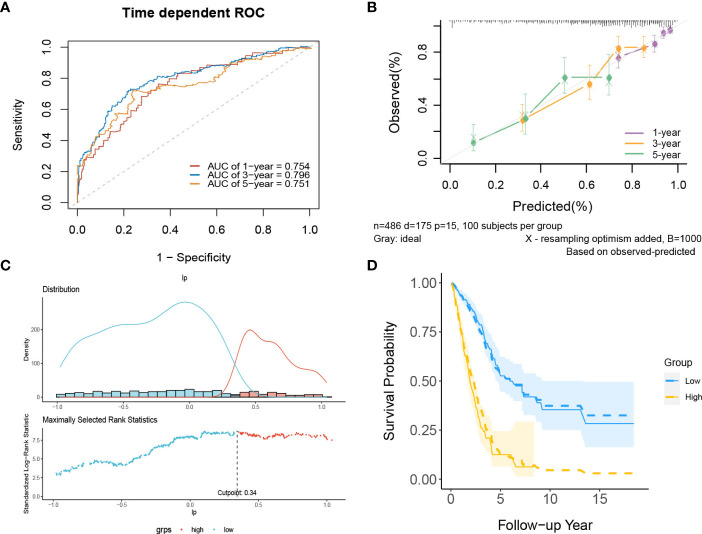
The performance of the lncRNA model in the derivation dataset. **(A)** 1-,3-,5-year ROC curves and **(B)** calibration plot of the lncRNA model. **(C)** The optimal cutoff value of PI. **(D)** Predicted versus observed survival probability in each risk group. Solid line: observed Kaplan-Meier curve; dotted line: average predicted survival curve; shaded area: 95% confidence interval of observed survival probability.

In internal validation, the apparent C-index was 0.730(95%CI:0.688-0.772), and the optimism-corrected C-index with 1000 bootstrap replications was 0.707. The AUCs of the model at 1, 3 and 5 years were 0.754, 0.796, and 0.751, respectively (**Figure 9A**). The calibration plot shows that the model has good agreement between predicted and observed survival probabilities at 1, 3 and 5 years (**Figure 9B**). Furthermore, patients were divided into two risk groups based on the optimal truncation value of PI (**Figure 9C**). It was further found that the observed Kaplan–Meier survival curves in the two risk groups were close to the average predicted survival curves (**Figure 9D**), which also proved that our prediction model had good calibration accuracy. **Figure S8** shows the Kaplan–Meier survival curves (**Figure S8A**) and risk factor association diagrams (**Figure S8B**) for the two risk groups.

**Figure 10:**
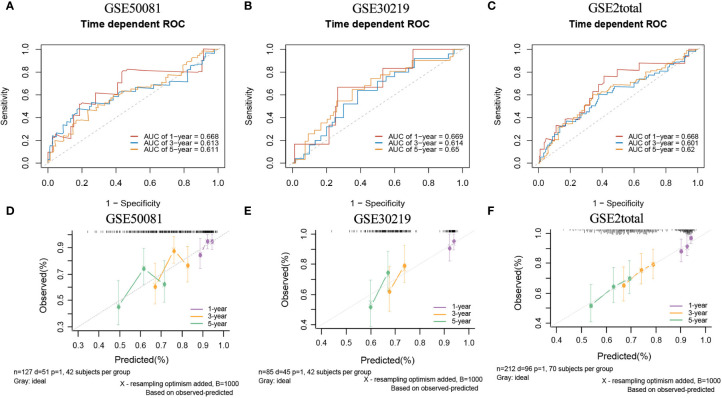
The ROC curves and calibration plots of lncRNA model in external validation data sets. ROC curves at 1-,3-,5-year: **(A)** GSE50081 dataset, **(B)** GSE30219 dataset, and **(C)** GSE2total dataset. The calibration plots at 1-,3-,5-year: **(D)** GSE50081 dataset, **(E)** GSE30219 dataset, and **(F)** GSE2total dataset.

In the external validation cohorts, three C indexes of the model were 0.596(95%CI:0.506-0.685)(GSE50081), 0.602(95%CI:0.525-0.682)(GSE30219) and 0.596(95%CI:0.534-0.658)(GSE2total). In addition, **Figure S4B** shows C-indexes of the model over 1-10 years in four datasets. Although C-indexes of the model in the validation set is lower than derivation set, they remained at 0.6 during the decade. According to the time-dependent ROC curves (**Figures 10A–C**), the area under the curves of the model in the three validation sets of 1, 3 and 5 years were all above 0.6. **Figures 10D–F** shows the calibration accuracy of the model in three external verification sets. Patients in the validation sets were divided into two risk groups based on the maximum rank statistics (**Figures S9A–C**), and the average predicted survival curves and observed survival curves in the two groups were compared to further validate the calibration accuracy of the model (**Figures S9D–F**). Unfortunately, the external validation calibration accuracy of the lncRNA model was not as ideal as that of mRNA model, but the prediction results within three years were close to the observations and did not deviate too far from reality within five years. Subsequently, the Kaplan–Meier survival curves of the two risk groups and the risk factor association diagrams of the model in each validation set are shown in **Figures S10, S11** respectively.

### Development and Validation of the Comprehensive Prediction Model

The prognostic indexes of the two gene models were used as candidate predictors, and the comprehensive prediction model was constructed by stepwise regression combined with three clinical variables (age, sex and tumor stage) to predict OS. The final model included three predictors: mRNA risk score, lncRNA risk score, and tumor stage. Based on the coefficients and predictors obtained from all imputed datasets, the final PI is structured as:


$$\begin{array}{l} \text{PI}=-0.3295+0.6015\times \text{mRNA Risk Score}+0.4540\times \text{IncRNA Risk Score }+\\ \text{tumor stage} \end{array}$$

in which:

Tumor stage: stage I=0, stage II= 0.6567, stage III= 0.7510, stage IV= 0.9675

The distribution of PI in the development data set and validation set is shown in **Figure S2C**. The base survival probability of the comprehensive prediction model from 1 to 10 years is also given in **Table S2**.

**Figure 11:**
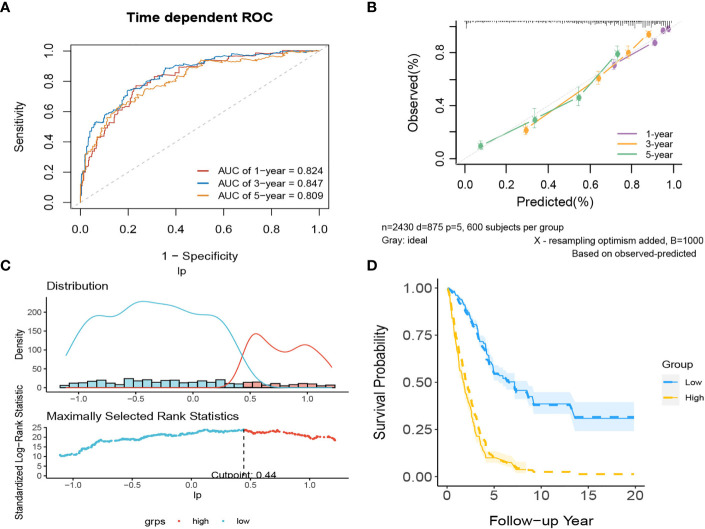
The performance of the comprehensive model in the derivation dataset. **(A)** 1-,3-,5-year ROC curves and **(B)** calibration plot of the comprehensive model. **(C)** The optimal cutoff value of PI. **(D)** Predicted versus observed survival probability per risk group.

In internal validation, the apparent C-index was 0.795(95%CI:0.780-0.810) the optimism-corrected C-index with 1000 bootstrap replications was 0.794. The 1-year, 3-year and 5-year AUCs of this model were 0.824, 0.847, and 0.809, respectively (**Figure 11A**). The calibration plot shows that the model has good agreement between predicted and observed survival probabilities at 1, 3 and 5 years (**Figure 11B**). Again, patients were divided into two risk groups based on the optimal truncation value of PI (**Figure 11C**). The observed Kaplan–Meier survival curves for the two risk groups almost overlap with the average predicted survival curves shown in **Figure 11D**, further confirming that the prediction model has good calibration accuracy in the derivation set.

**Figure 12:**
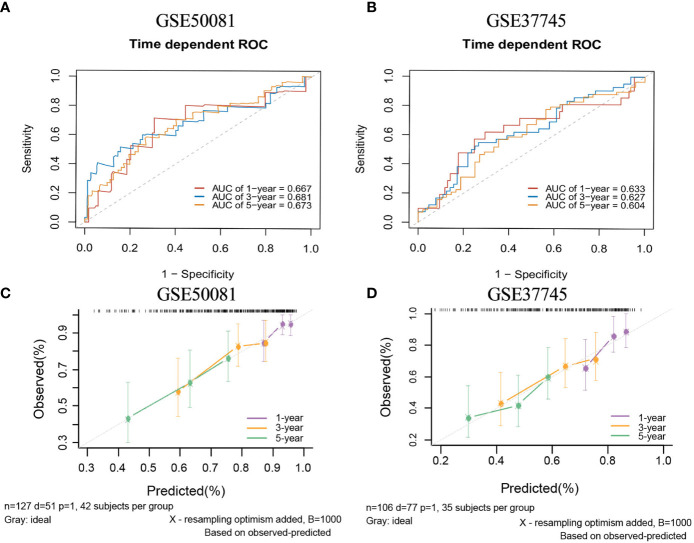
The ROC curves and calibration plots of comprehensive model in external validation data sets.1-,3-,5-year ROC curves: **(A)** GSE50081 dataset and **(B)** GSE37745 dataset. The calibration plots: **(C)** GSE50081 dataset and **(D)** GSE37745 dataset.

There are two data sets used as external validation sets for this model. In the external validation cohorts, the two C indexes of the model were 0.649(95%CI:0.564-0.733)(GSE50081) and 0.606(95%CI:0.536-0.677) (GSE37745). **Figure S4C** shows the C-index of the model over 1-10 years in the three datasets. **Figures 12A–B** shows the ROC curve of the model in the two validation sets, and **Figures 12C–D** shows the calibration plots. Again, we divided samples into two risk groups (**Figures S12A, B**) and then compared the observed survival curves in the two risk groups with the average predicted survival curves (**Figures S12C, D**). In GSE50081, the model still has the risk of underestimating the survival probability (**Figure S12C**). However, in GSE37745, the predicted average survival probability curves were quite close to the actual curve, showing very good consistency (**Figure S12D**).

From this model, we created a nomogram to predict the prognostic survival probability of patients with lung adenocarcinoma at 1, 3 and 5 years (**Figure S13**). Subsequently, we used decision curve analysis (DCA) to compare and demonstrate the net benefits of the clinical utility of the three models at 1, 3 and 5 years (**Figure S14**). With increasing time, the net benefits of the three models continued to increase, and the net benefits of the mRNA model and lncRNA model at the three time points showed little difference. As a matter of course, the net benefit of the comprehensive model is always the greatest.

### Online Calculators for Models

To facilitate the clinical application of the model, the three model calculations mentioned in this paper can be completed by this website: https://lhj0520.shinyapps.io/LUAD_prediction_model/. Enter or select the value of the variable and the time you want to predict in the gray box on the left side of the page and then click the “forecast” button at the bottom to obtain the corresponding point estimate or survival curve on the right side (**Figure S15**).

### Drug Prediction and TIDE Immunotherapy Prediction Analyses

**Figure 13:**
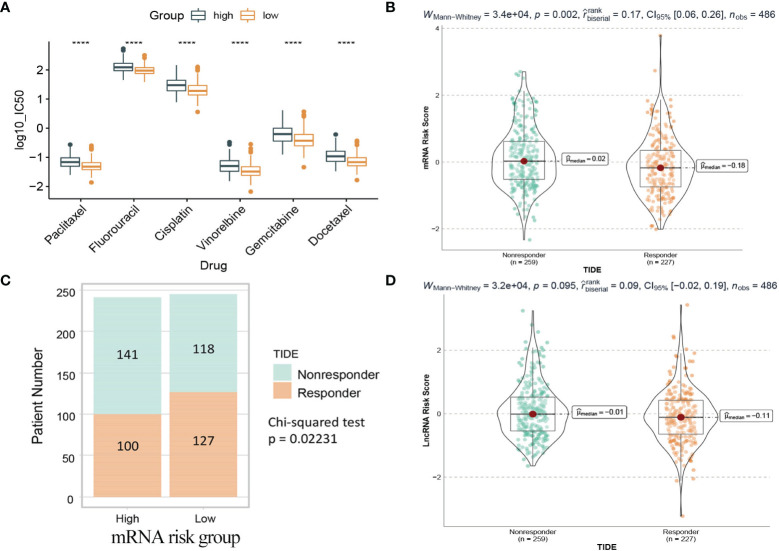
Drug prediction and TIDE immunotherapy prediction analyses. **(A)** Box plot of IC50 values of six chemotherapy drugs between the two risk groups in the mRNA model. **(B)** The mRNA risk score between TIDE predicted responders and non-responders. **(C)** Distribution of TIDE responders and non-responders in the mRNA risk groups. **(D)** The lncRNA risk score between TIDE predicted responders and non-responders. Responder: the patient who responds to the immune checkpoint inhibitors. Nonresponder: the patient who does not respond to the immune checkpoint inhibitors. ****: <0.0001.

Chemotherapy plays a critical role in curing or controlling lung adenocarcinoma. The IC50 estimates of 6 common chemotherapeutic drugs were calculated from the GDSC database. The difference of IC50 between the high and low risk groups in the mRNA model was compared. The results (**Figure 13A**) showed that the IC50 values of all 6 drugs were significantly different between the high-risk group and the low-risk group, and patients in the low-risk group were more sensitive than the high-risk group.

Immunotherapy using immune checkpoint inhibitors has brought hope to LUAD patients. The response of 486 patients in the TCGA dataset to immune checkpoint inhibitors was calculated based on the gene expression matrix through the TIDE website. As shown in **Figure 13B**, for the mRNA model, the risk score of patients in the nonresponse group (n=259) was higher than that in the response group (n=227), and the difference was statistically significant (Wilcoxon test, p=0.002). Further analysis (**Figure 13C**) showed that patients in the low-risk group (127/245) were more sensitive to immunotherapy than those in the high-risk group (100/241). In contrast, in the lncRNA model, the difference was not statistically significant (Wilcoxon test, p=0.095), so it could not be considered that there was a difference in risk scores between the two groups (**Figure 13D**).

### Study of Somatic Variation in the mRNA Model

**Figure 14:**
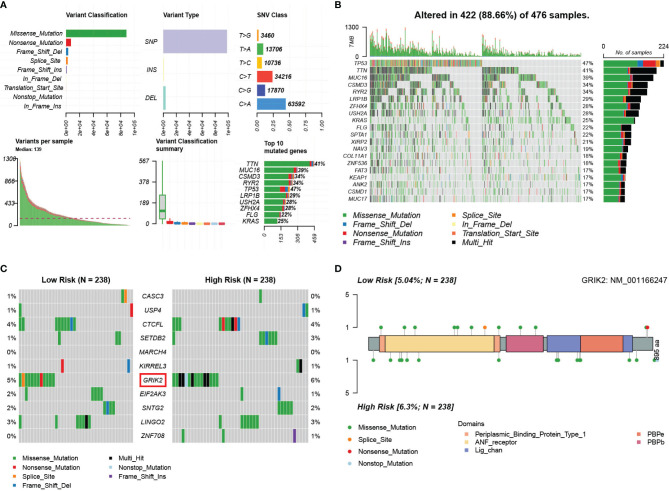
Landscape of somatic mutations in lung adenocarcinoma patients in TCGA. **(A)** the summary of the mutation data. **(B)** The waterfall plot of the mutation distribution of the top 20 most frequently mutated genes. **(C)** The waterfall plot of the mutation distribution of 11 predictors between two risk groups in the mRNA model. **(D)** The lollipop pot of the differential distribution of variants of GRIK2 between two risk groups in mRNA model.

We obtained single nucleotide mutations data for 476 LUAD patients (ten samples were not available) from the GDC Data Portal. **Figure 14A** is a summary of the mutation data. More detailed mutation information is shown in **Figure 14B**. Different colors represent different types of mutations. In addition, we compared the mutations in genes in the mRNA model between the two risk groups (**Figure 14C**). *GRIK2* was found to be the mutated gene with the most common frequency in both groups, which mutated more in the high-risk group (**Figure 14D**). More intriguingly, we calculated co-occurrence and mutually exclusive mutations between 11 genes and found only two group co-occurrence mutations, including *GRIK2*(**Figure S16A**). Subsequently, we plotted the mutation frequency of genes into gene word clouds, as shown in **Figure S16B**. Further, we calculated the tumor mutation burden (TMB) in 476 samples (**Figure S16C**). We compared the TMB of the responder and non-responder groups in TIDE. The TMB of the responder group was higher than that of the non-responder group (Wilcoxon test, p=0.028, **Figure S16D**), indicating that patients with higher TMB may have a better effect on immunotherapy.

## Discussion

Commonly used predictive models for lung adenocarcinoma based on m^6^A methylated relevant genes have been developed, but these models are not yet complete in terms of application. This study constructed clinical prediction models at three different levels based on m^6^A-related mRNAs, lncRNAs and clinical information data, and collected multiple external validation sets for validation. We reported this study according to the Transparent Reporting of a Multivariable Prediction Model for Individual Prognosis or Diagnosis Statement (TRIPOD). The complete checklist is shown in **Table S3**.

The first model was developed based on m^6^A-related mRNA and contained 11 genes in total (**Table S4**). Compared with other models, our model contains more genes. However, in several independent external validation sets, the model shows relatively stable and good discrimination and calibration. At present, studies have shown that *USP4*, *EIF2AK3* and *CTCFL* genes are related to the prognosis of lung adenocarcinoma (42–44).

The 11 genes are all obtained from m6Avar database (now updated to “RMVar”). Variants of these genes were hypothesized to affect RNA modifications (e.g., m^6^A) and thus disease (14). The m^6^A-associated variants of 11 genes came from three different confidence levels of sources and two aspects of modification function (**Figure S17**). Four of the mutations lead to lost m^6^A sites (*USP4, CTCFL, GRIK2, SNTG2*) and ten of the mutations lead to gain m^6^A sites (*ZNF708, LINGO2, EIF2AK3, KIRREL3, MARCH4, SETDB2, USP4, CASC3*). For m^6^A sites with high confidence level were derived from miCLIP or PA-m6A-seq experiments (3, 45, 46) and the three m^6^A-associated variants (*SETDB2, MARCH4, EIF2AK3*) were retained because of locating nearby the m^6^A sites or disrupting *DRACH* motif around the m^6^A sites (47–49). For m^6^A sites having a medium confidence level which were predicted from the previously published MeRIP-seq data (50–52), the four m^6^A-associated variants (*KIRREL3, EIF2AK3, ZNF708, LINGO2*) were derived from the intersection between the variants and the m^6^A sites generated from MeRIP-Seq experiments. For m^6^A sites with a low confidence level predicted by transcriptome-wide prediction, the seven m^6^A-associated variants (*CTCFL, GRIK2, SNTG2, CASC3, KIRREL3* and *USP4* have two variants) were predicted by the Random Forest prediction model (14). In addition, disease-related data from GWAS and ClinVar databases were collected to determine that the variants of 11 genes were pathogenic mutations leading to dysregulation of m^6^A modification in lung adenocarcinoma (14). Furthermore, we calculated the correlation coefficients between 11 genes and 21 m^6^A regulatory factors (**Figure S18**). It turns out that there are varying degrees of correlation between each predictor and regulator.

For mRNA risk score, we also explored their relationship with common chemotherapy drugs and immunotherapy. The study found that patients in the low-risk group were less resistant to commonly used chemotherapy drugs than those in the high-risk group. Furthermore, 11 mRNAs and risk score were calculated for their association with each chemotherapy drug (**Figure S19**). Risk scores were positively correlated with IC50 of all drugs (i.e., patients with higher scores had higher resistance to chemotherapy drugs), indicating that patients with higher scores were insensitive to chemotherapy. Five of the 11 mRNAs (*CTCFL, MARCH4, KIRREL3, GRIK2, LINGO2*) were also positively correlated with IC50 of all drugs. By analyzing the relationship between TIDE score and mRNA risk score, we found that patients with low TIDE scores were more likely to respond to immune checkpoint inhibitors. This may help predict the efficacy of immunotherapy for LUAD. In addition, it is currently believed that a higher value of tumor mutation load represents the higher immunogenicity of the tumor, which is more conducive to immunotherapy drugs, and our analysis also confirmed this view again.

The second model was constructed based on m^6^A related lncRNAs. There are 15 predictors in total (**Table S5**). The variables screening process of lncRNA model is relatively complex, and repeated exploration is to find a prediction model with relatively good discrimination. There are not enough studies on lncRNA in lung adenocarcinoma, but four at present: *SNHG12, RPARP-AS1, CRNDE, LMO7DN. SNHG12* has been experimentally predicted as a potential biomarker for the diagnosis, treatment and prognosis of LUAD (53). *RPARP-AS1* and *CRNDE* were included as two predictors in another literature (54). *LMO7DN* has also been suggested as a predictor of lung adenocarcinoma associated with ferroptosis (55).

The third model combined the risk scores of the first two models with clinical variable. There are 3 predictors in total: mRNA risk score, lncRNA risk score, tumor stage. We considered combination of prognostic indices of the two transcriptomic predictive models with clinical variables as a new approach to prognosis prediction and achieved good results.

This study has several advantages. First, all models are based on public cohort data from reliable sources that predict a long survival interval of up to 10 years. Each model was externally validated by multiple independent data sets and stable validation results were obtained. In addition, considering the usability of the model, a model-related web calculator has been developed for anyone to use.

There are several limitations to our study. First, when constructing the comprehensive model, we narrowed the candidate predictors in the development model to three (age, sex, and tumor stage), taking into account the fragmentary clinical variables in validation sets. But it also simplifies the final model somewhat. Secondly, the three models derived in this study are somewhat complicated. In order to reduce the difficulty of practical prediction caused by complex and diverse models, we developed a web calculator containing all models. Thirdly, the performance of our model in external verification will take into account the difference between verification set and derivation set. If the difference is too large, our model may not achieve good performance.

In conclusion, we developed and externally validated three models to predict survival probability of lung adenocarcinoma based on m^6^A-related transcriptomics. This may provide clues to new strategies or therapeutic targets for lung adenocarcinoma.

## Data Availability Statement

Publicly available datasets were analyzed in this study. This data can be found here: http://xena.ucsc.edu/. https://www.ncbi.nlm.nih.gov/geo/.

## Author Contributions

HL, S-BL, and JS contributed to conceptualization and project administration. HL, S-BL, JC, and NY downloaded and analyzed the data. HL, SB-L wrote the manuscript, with assistance from JS, LB, XZ, KL, and ZT. All authors reviewed the manuscript.

## Funding

This work was supported in part by the National Natural Science Foundation of China (81773541), funded from the Priority Academic Program Development of Jiangsu Higher Education Institutions at Soochow University, the State Key Laboratory of Radiation Medicine and Protection (GZK1201919) to ZT, National Natural Science Foundation of China (81872552, U1967220) to JC. Science and Technology Innovation team project of the Suzhou Vocational health college (SZWZYTD201804), Qing-Lan Project of Jiangsu Province in China (2021) to S-BL. National Natural Science Foundation of China (82172441), Suzhou Key Clinical Diagnosis and Treatment Technology Project (LCZX201925) to KL.

## Conflict of Interest

The authors declare that the research was conducted in the absence of any commercial or financial relationships that could be construed as a potential conflict of interest.

## Publisher’s Note

All claims expressed in this article are solely those of the authors and do not necessarily represent those of their affiliated organizations, or those of the publisher, the editors and the reviewers. Any product that may be evaluated in this article, or claim that may be made by its manufacturer, is not guaranteed or endorsed by the publisher.
