# Supplementary material for: Development and Validation of Prognostic Model for Lung Adenocarcinoma Patients Based on m6A Methylation Related Transcriptomics
Source: Front Oncol. 2022 Jun 16;12:895148. doi: 10.3389/fonc.2022.895148 (PMC9243308; doi:10.3389/fonc.2022.895148)
Supplement: Supplementary file 1 [file DataSheet_1.docx]

**Supplementary data**

Table S1. 21 m^6^A methylation regulators from the literature

| Ensembl ID | Regulaors | Full name | Type |
| --- | --- | --- | --- |
| ENSG00000165819 | METTL3 | methyltransferase like 3 | writers |
| ENSG00000145388 | METTL14 | methyltransferase like 14 | writers |
| ENSG00000162775 | RBM15 | RNA binding motif protein 15 | writers |
| ENSG00000259956 | RBM15B | RNA binding motif protein 15B | writers |
| ENSG00000146457 | WTAP | WT1 associated protein | writers |
| ENSG00000164944 | KIAA1429 | Vir like m6A methyltransferase associated | writers |
| ENSG00000105879 | CBLL1 | Cbl proto-oncogene like 1 | writers |
| ENSG00000123200 | ZC3H13 | zinc finger CCCH-type containing 13 | writers |
| ENSG00000091542 | ALKBH5 | alkB homolog 5, RNA demethylase | erasers |
| ENSG00000140718 | FTO | FTO, alpha-ketoglutarate dependent dioxygenase | erasers |
| ENSG00000083896 | YTHDC1 | YTH domain containing 1 | readers |
| ENSG00000047188 | YTHDC2 | YTH domain containing 2 | readers |
| ENSG00000149658 | YTHDF1 | YTH N6-methyladenosine RNA binding protein 1 | readers |
| ENSG00000198492 | YTHDF2 | YTH N6-methyladenosine RNA binding protein 2 | readers |
| ENSG00000185728 | YTHDF3 | YTH N6-methyladenosine RNA binding protein 3 | readers |
| ENSG00000159217 | IGF2BP1 | insulin like growth factor 2 mRNA binding protein 1 | readers |
| ENSG00000122566 | HNRNPA2B1 | heterogeneous nuclear ribonucleoprotein A2/B1 | readers |
| ENSG00000092199 | HNRNPC | heterogeneous nuclear ribonucleoprotein C (C1/C2) | readers |
| ENSG00000102081 | FMR1 | fragile X mental retardation 1 | readers |
| ENSG00000138095 | LRPPRC | leucine rich pentatricopeptide repeat containing | readers |
| ENSG00000066044 | ELAVL1 | ELAV like RNA binding protein 1 | readers |

Table S2. Baseline survival probabilities ($S_{0}(t)$) of each model

| Year | Baseline survival probabilities | | |
| --- | --- | --- | --- |
|  | mRNA model | lncRNA model | comprehensive model |
| 1 | 0.886285 | 0.885123219 | 0.886964098 |
| 2 | 0.767485 | 0.759435906 | 0.769773728 |
| 3 | 0.649213 | 0.631038554 | 0.649685955 |
| 4 | 0.537818 | 0.517947805 | 0.533572166 |
| 5 | 0.430639 | 0.409327909 | 0.422790676 |
| 6 | 0.405304 | 0.383556056 | 0.396601941 |
| 7 | 0.385595 | 0.362784709 | 0.375921810 |
| 8 | 0.345159 | 0.322255278 | 0.332077064 |
| 9 | 0.323665 | 0.300128827 | 0.305686059 |
| 10 | 0.300341 | 0.275445652 | 0.278333157 |

Table S3 Transparent Reporting of a multivariable prediction model for Individual Prognosis or Diagnosis statement Checklist

| **Section/Topic** | **Item** |  | **Checklist Item** | **Page** |
| --- | --- | --- | --- | --- |
| **Title and abstract** | | | | |
| Title | 1 | D;V | Identify the study as developing and/or validating a multivariable prediction model, the target population, and the outcome to be predicted. | 1 |
| Abstract | 2 | D;V | Provide a summary of objectives, study design, setting, participants, sample size, predictors, outcome, statistical analysis, results, and conclusions. | 2 |
| **Introduction** | | | | |
| Background and objectives | 3a | D;V | Explain the medical context (including whether diagnostic or prognostic) and rationale for developing or validating the multivariable prediction model, including references to existing models. | 3 |
|  | 3b | D;V | Specify the objectives, including whether the study describes the development or validation of the model or both. | 3 |
| **Methods** | | | | |
| Source of data | 4a | D;V | Describe the study design or source of data (e.g., randomized trial, cohort, or registry data), separately for the development and validation data sets, if applicable. | 3-4 |
|  | 4b | D;V | Specify the key study dates, including start of accrual; end of accrual; and, if applicable, end of follow-up. | Not applicable |
| Participants | 5a | D;V | Specify key elements of the study setting (e.g., primary care, secondary care, general population) including number and location of centres. | Not applicable |
|  | 5b | D;V | Describe eligibility criteria for participants. | 3-4 |
|  | 5c | D;V | Give details of treatments received, if relevant. | Not applicable |
| Outcome | 6a | D;V | Clearly define the outcome that is predicted by the prediction model, including how and when assessed. | Not applicable |
|  | 6b | D;V | Report any actions to blind assessment of the outcome to be predicted. | Not applicable |
| Predictors | 7a | D;V | Clearly define all predictors used in developing or validating the multivariable prediction model, including how and when they were measured. | 4 |
|  | 7b | D;V | Report any actions to blind assessment of predictors for the outcome and other predictors. | Not applicable |
| Sample size | 8 | D;V | Explain how the study size was arrived at. | 3-4 |
| Missing data | 9 | D;V | Describe how missing data were handled (e.g., complete-case analysis, single imputation, multiple imputation) with details of any imputation method. | 6 |
| Statistical analysis methods | 10a | D | Describe how predictors were handled in the analyses. | 4-6 |
|  | 10b | D | Specify type of model, all model-building procedures (including any predictor selection), and method for internal validation. | 4-6 |
|  | 10c | V | For validation, describe how the predictions were calculated. | Not applicable |
|  | 10d | D;V | Specify all measures used to assess model performance and, if relevant, to compare multiple models. | 4-6 |
|  | 10e | V | Describe any model updating (e.g., recalibration) arising from the validation, if done. | Not applicable |
| Risk groups | 11 | D;V | Provide details on how risk groups were created, if done. | 5 |
| Development vs. validation | 12 | V | For validation, identify any differences from the development data in setting, eligibility criteria, outcome, and predictors. | Not applicable |
| **Results** | | | | |
| Participants | 13a | D;V | Describe the flow of participants through the study, including the number of participants with and without the outcome and, if applicable, a summary of the follow-up time. A diagram may be helpful. | Not applicable |
|  | 13b | D;V | Describe the characteristics of the participants (basic demographics, clinical features, available predictors), including the number of participants with missing data for predictors and outcome. | 7 |
|  | 13c | V | For validation, show a comparison with the development data of the distribution of important variables (demographics, predictors and outcome). | Table 1 |
| Model development | 14a | D | Specify the number of participants and outcome events in each analysis. | Table 1 |
|  | 14b | D | If done, report the unadjusted association between each candidate predictor and outcome. | Not applicable |
| Model specification | 15a | D | Present the full prediction model to allow predictions for individuals (i.e., all regression coefficients, and model intercept or baseline survival at a given time point). | Table S2 |
|  | 15b | D | Explain how to the use the prediction model. | 8 |
| Model performance | 16 | D;V | Report performance measures (with CIs) for the prediction model. | 7-11 |
| Model-updating | 17 | V | If done, report the results from any model updating (i.e., model specification, model performance). | Not applicable |
| **Discussion** | | | | |
| Limitations | 18 | D;V | Discuss any limitations of the study (such as nonrepresentative sample, few events per predictor, missing data). | 13 |
| Interpretation | 19a | V | For validation, discuss the results with reference to performance in the development data, and any other validation data. | Not applicable |
|  | 19b | D;V | Give an overall interpretation of the results, considering objectives, limitations, results from similar studies, and other relevant evidence. | 12-13 |
| Implications | 20 | D;V | Discuss the potential clinical use of the model and implications for future research. | 13-14 |
| **Other information** | | | | |
| Supplementary information | 21 | D;V | Provide information about the availability of supplementary resources, such as study protocol, Web calculator, and data sets. | Supplement and online calculator |
| Funding | 22 | D;V | Give the source of funding and the role of the funders for the present study. | 15 |

Table S4. 11 genes in mRNA model

| Ensembl ID | Gene | Full name |
| --- | --- | --- |
| ENSG00000108349 | CASC3 | CASC3 exon junction complex subunit |
| ENSG00000114316 | USP4 | ubiquitin specific peptidase 4 |
| ENSG00000124092 | CTCFL | CCCTC-binding factor like |
| ENSG00000136169 | SETDB2 | SET domain bifurcated histone lysine methyltransferase 2 |
| ENSG00000144583 | MARCH4 | membrane associated ring-CH-type finger 4 |
| ENSG00000149571 | KIRREL3 | kirre like nephrin family adhesion molecule 3 |
| ENSG00000164418 | GRIK2 | glutamate ionotropic receptor kainate type subunit 2 |
| ENSG00000172071 | EIF2AK3 | eukaryotic translation initiation factor 2 alpha kinase 3 |
| ENSG00000172554 | SNTG2 | syntrophin gamma 2 |
| ENSG00000174482 | LINGO2 | leucine rich repeat and Ig domain containing 2 |
| ENSG00000182141 | ZNF708 | zinc finger protein 708 |

Table S5. 15 lncRNAs in lncRNA model

| Ensembl ID | Gene | Full name |
| --- | --- | --- |
| ENSG00000197989 | SNHG12 | small nucleolar RNA host gene 12 |
| ENSG00000269609 | RPARP-AS1 | C10orf95 antisense RNA 1 |
| ENSG00000245694 | CRNDE | colorectal neoplasia differentially expressed |
| ENSG00000178734 | LMO7DN | LMO7 downstream neighbor |
| ENSG00000249476 | AC008467.1 | uncharacterized LOC285638 |
| ENSG00000259070 | LINC00639 | long intergenic non-protein coding RNA 639 |
| ENSG00000242686 | AC107464.1 | PDE6B antisense RNA 1 |
| ENSG00000235138 | AL445931.1 | uncharacterized LOC100130548 |
| ENSG00000237975 | FLG-AS1 | FLG antisense RNA 1 |
| ENSG00000224186 | C5orf66 | PITX1 antisense RNA 1 |
| ENSG00000245522 | AC026250.1 | long intergenic non-protein coding RNA 2709 |
| ENSG00000232527 | AC245595.1 | long intergenic non-protein coding RNA 2802 |
| ENSG00000254226 | LINC01933 | long intergenic non-protein coding RNA 1933 |
| ENSG00000233621 | LINC01137 | ZC3H12A divergent transcript |
| ENSG00000225855 | RUSC1-AS1 | RUSC1 antisense RNA 1 |


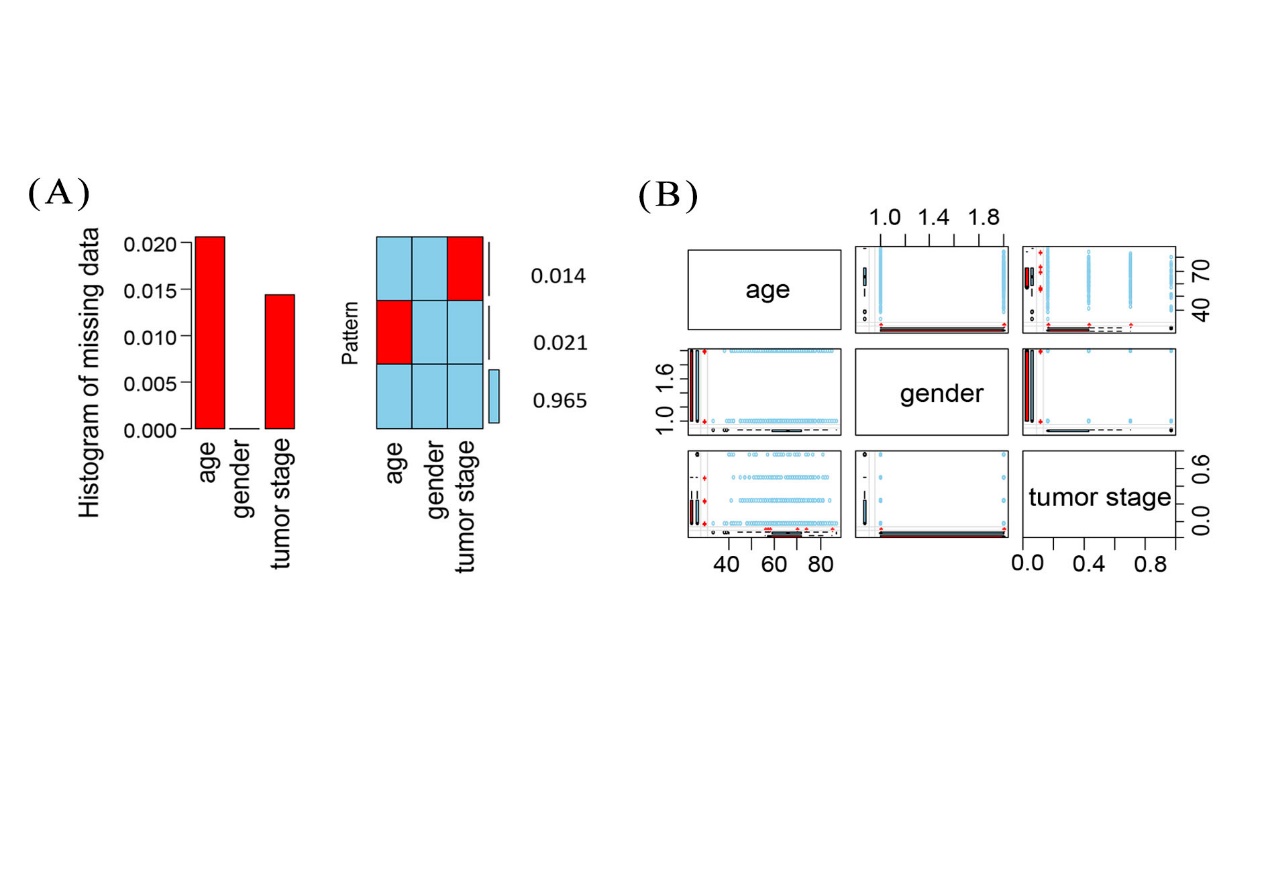


**Supplementary Figure 1**  Missing data in the TCGA dataset. (A) Calculate and plot the number of missing values in each clinical variable. (B) Create a scatterplot matrix with information about missing values in the plot margins of each panel. Blue: observed values. Red: missing values. The vertical box plots represent the variables on the vertical axis and the horizontal box plots represent the variables on the horizontal axis.


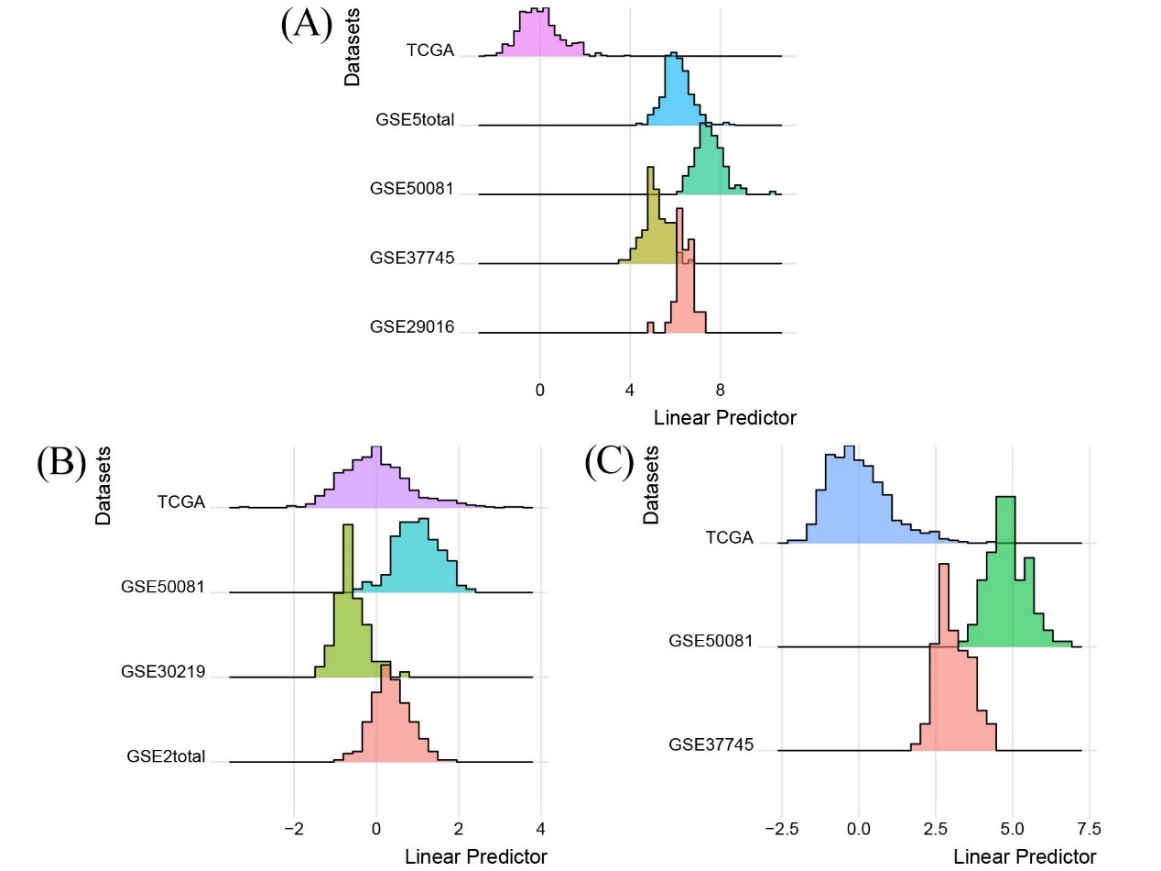


**Supplementary Figure 2** Distribution of linear predictors for (A)the mRNA model in five datasets, (B)the lncRNA model in four datasets, and (C) the comprehensive model in three datasets, respectively.


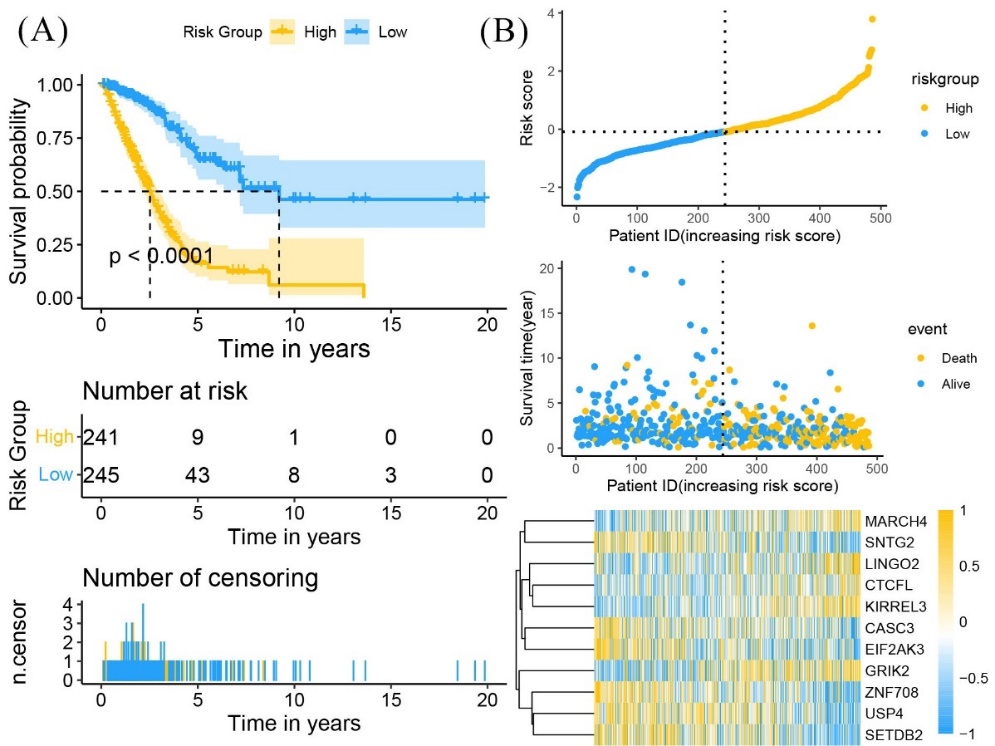


**Supplementary Figure 3**  The two risk groups of the mRNA model in the TCGA dataset. (A) The Kaplan-Meier curve of the two groups. (B) Risk factor association diagram of the model in the TCGA dataset. From top to bottom, the distribution of risk score, survival status of patients, and the heat map of expression of 11 genes in TCGA, respectively.


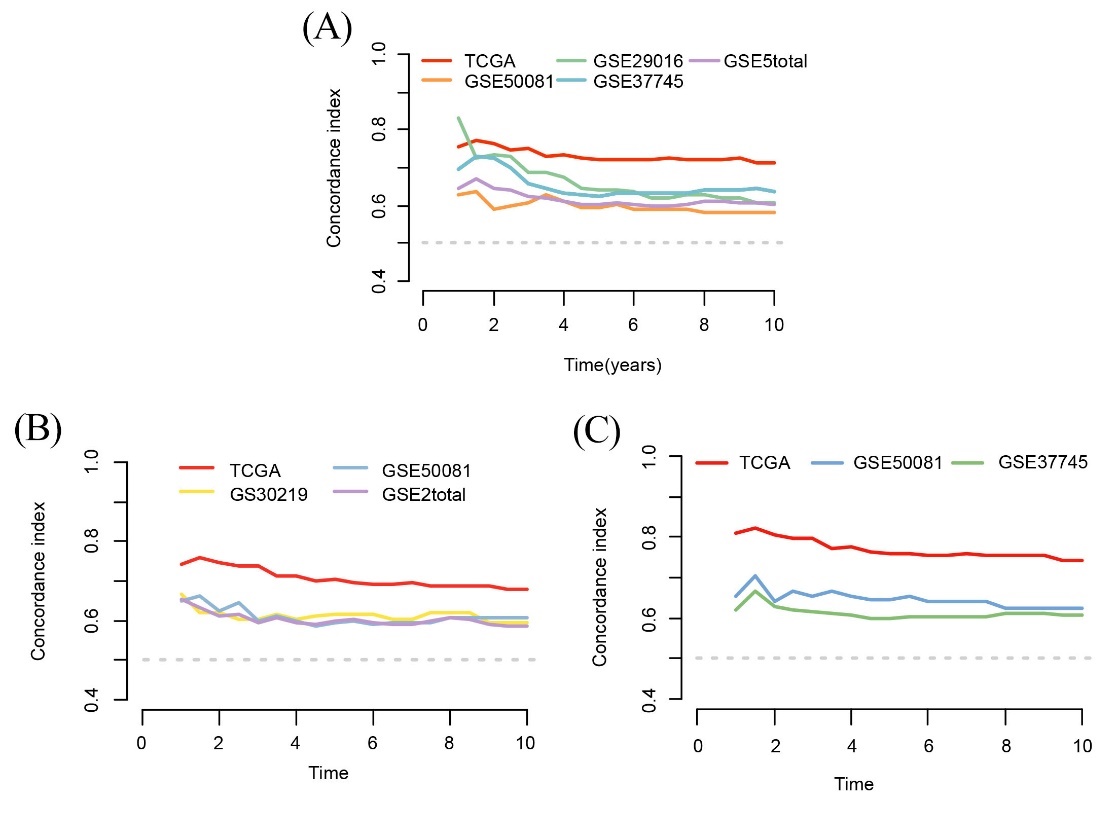


**Supplementary Figure 4 T**he time-dependent C-indexes of (A) the mRNA model in 5 data sets, (B) the lncRNA model in 4 data sets, and (C) the comprehensive model in 3 data sets, respectively.


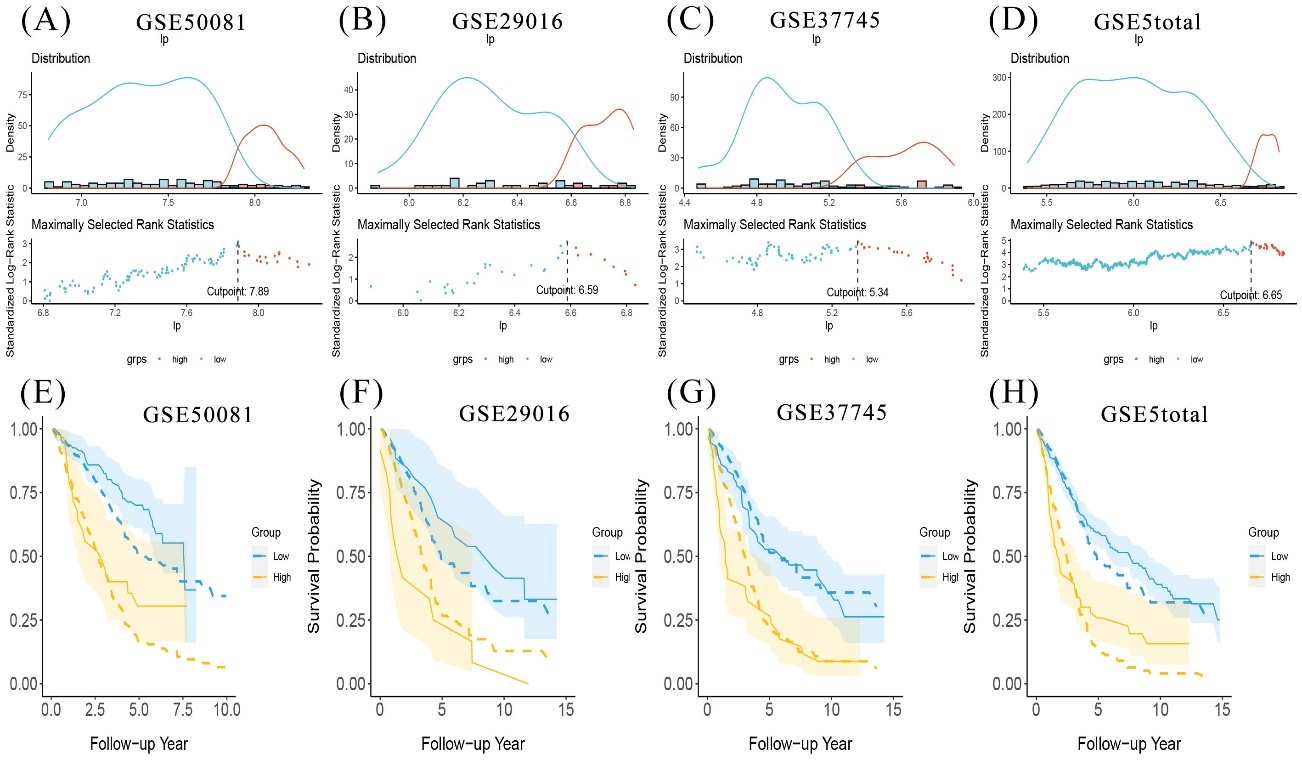


**Supplementary Figure 5** The calibration accuracy of mRNA model in external validation data sets. The optimal cutoff value of PI: (A) GSE50081 dataset, (B) GSE29016 dataset, (C) GSE37745 dataset, and (D) GSE5total dataset. Predicted versus observed survival probability in per risk group: (E) GSE50081 dataset, (F) GSE29016 dataset, (G) GSE37745 dataset, and (H) GSE5total dataset.


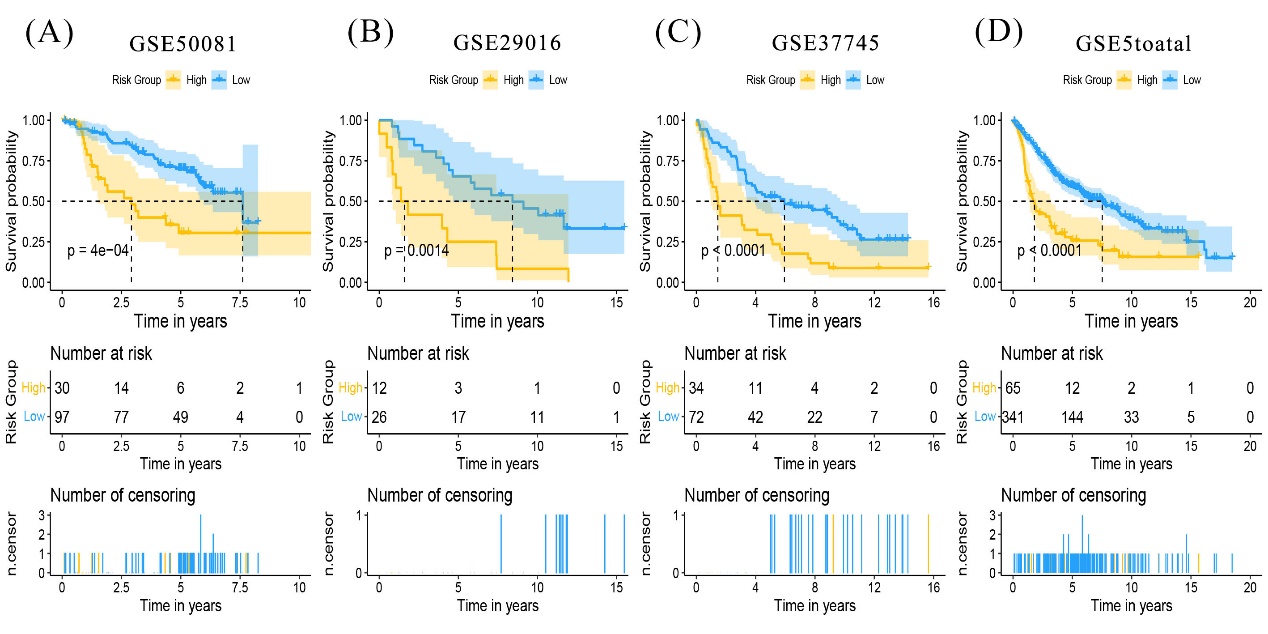


**Supplementary Figure 6** The Kaplan-Meier curve of the two risk groups in mRNA model in external validation data sets. (A) GSE50081 dataset. (B) GSE29016 dataset. (C) GSE37745 dataset. (D) GSE5total dataset.


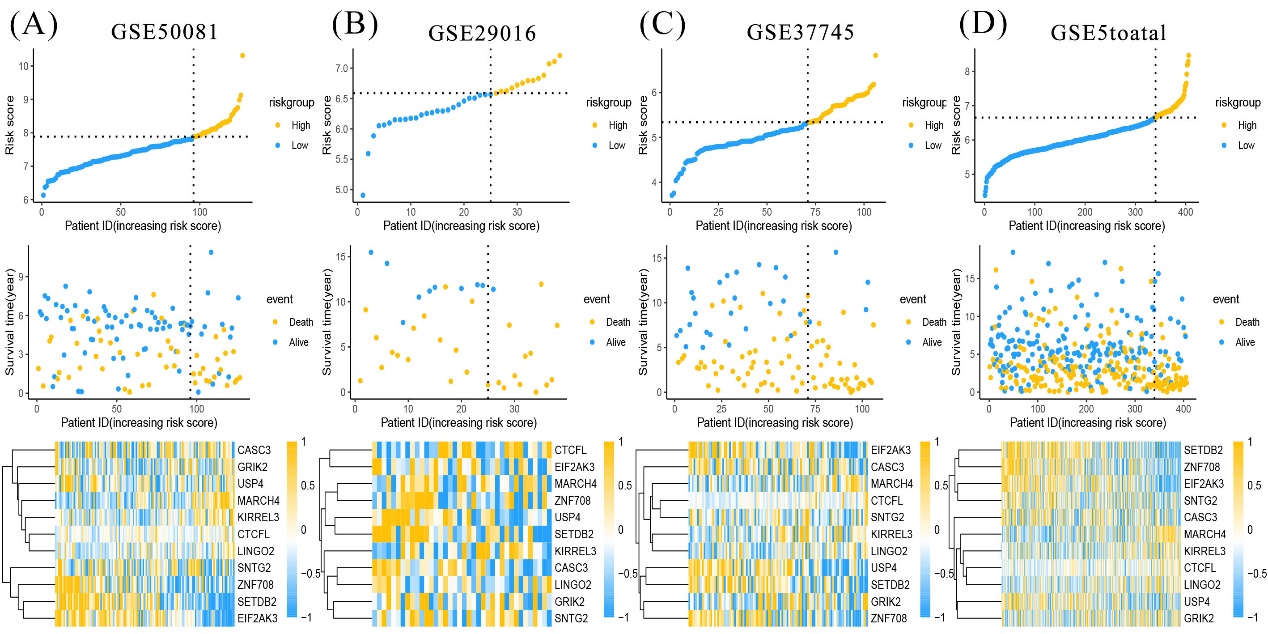


**Supplementary Figure 7** The risk factor association diagram of the mRNA model in external validation data sets. (A) GSE50081 dataset. (B) GSE29016 dataset. (C) GSE37745 dataset. (D) GSE5total dataset.


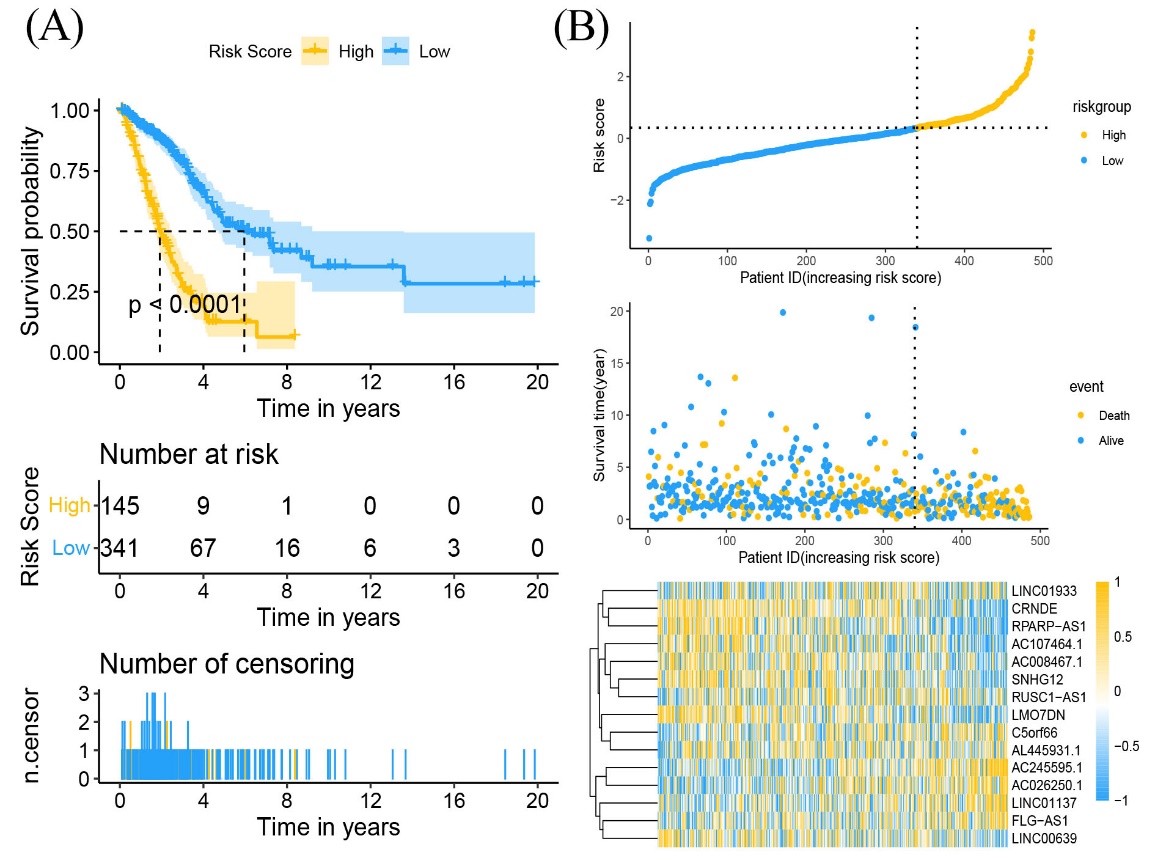


**Supplementary Figure 8** The two risk groups of lncRNA model in the TCGA dataset. (A) The Kaplan-Meier curve of the two groups. (B) Risk factor association diagram of the model. From top to bottom, the distribution of risk score, survival status of patients, and the heat map of expression of 15 lncRNAs, respectively.


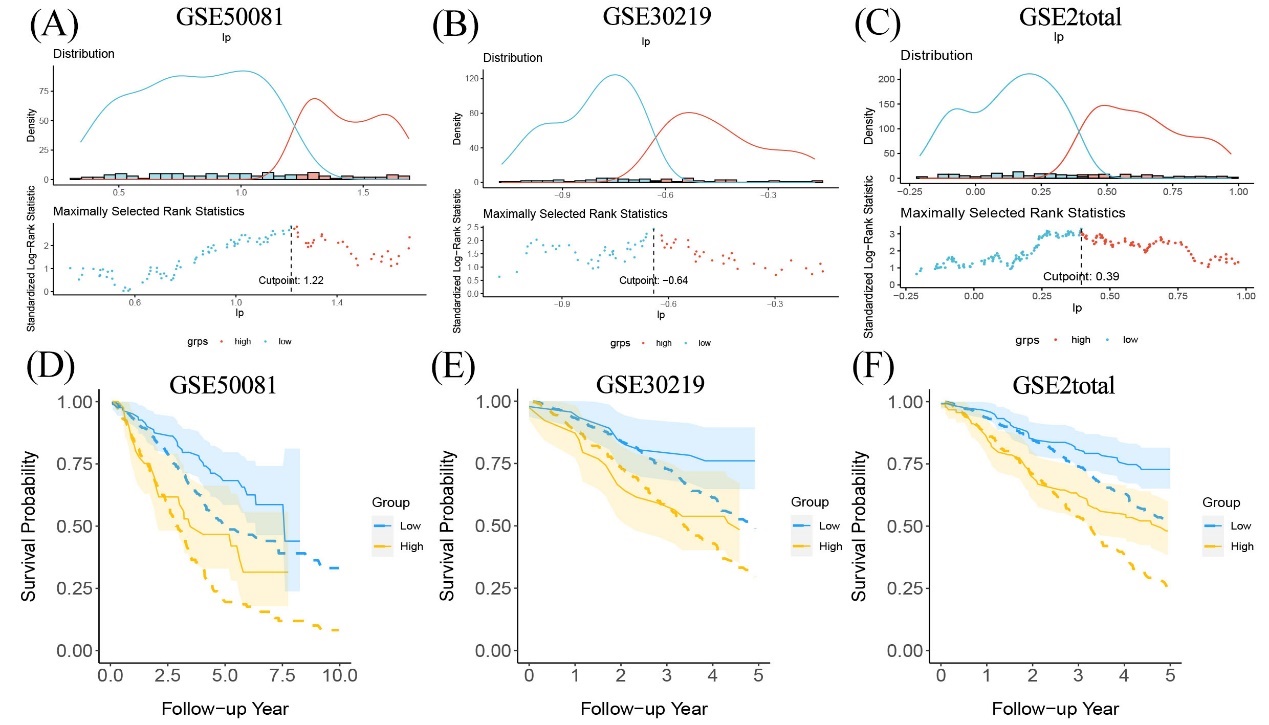


**Supplementary Figure 9** The calibration accuracy of lncRNA model in external validation data sets. The optimal cutoff value of PI: (A) GSE50081 dataset, (B) GSE30219 dataset, and (C) GSE2total dataset. Predicted versus observed survival probability in two risk groups : (D) GSE50081 dataset, (E) GSE30219 dataset, and (F) GSE2total dataset.


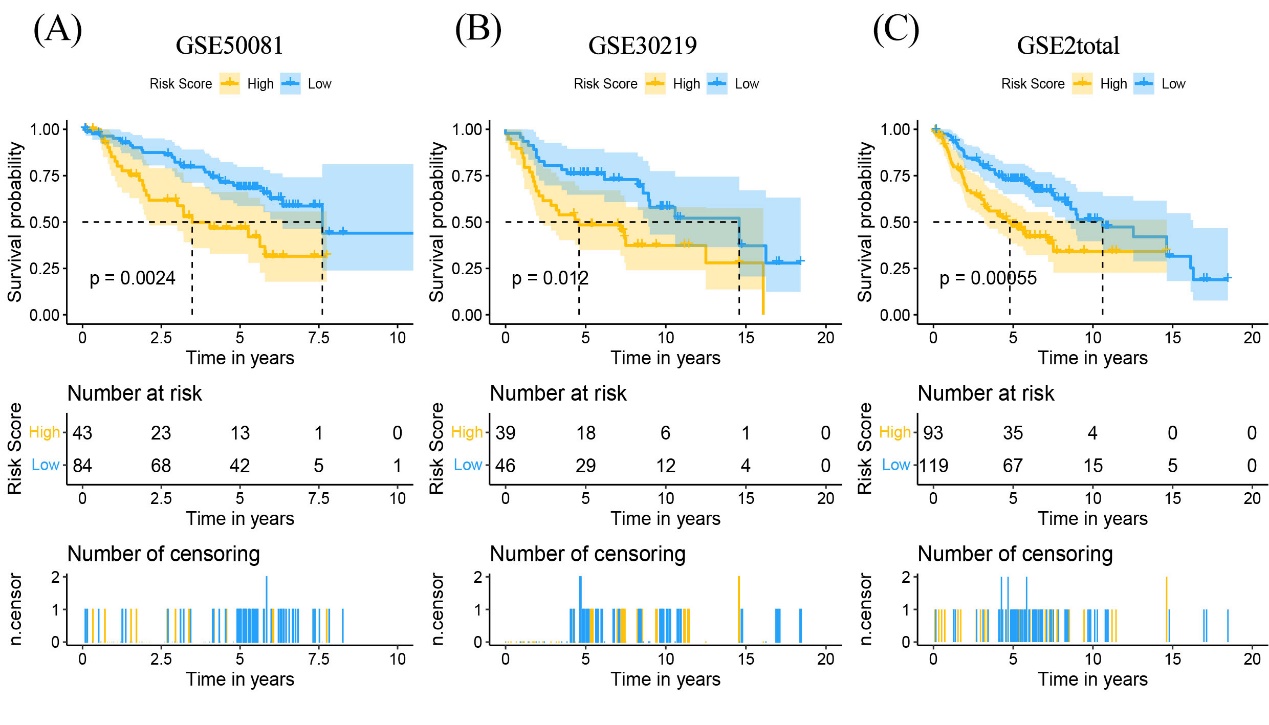


**Supplementary Figure 10** The Kaplan-Meier curve of the two risk groups in lncRNA model in external validation data sets. (A) GSE50081 dataset. (B) GSE30219 dataset. (C) GSE2total dataset.


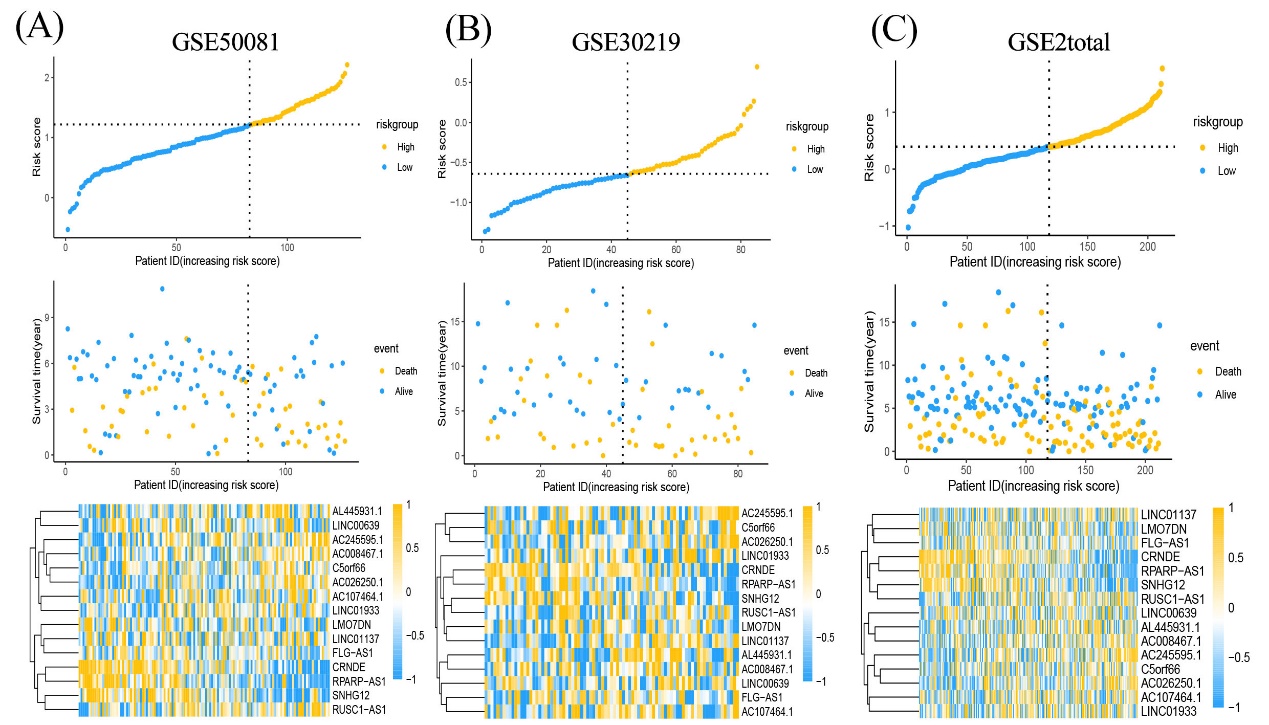


**Supplementary Figure 11** The risk factor association diagram of the lncRNA model in external validation data sets. (A) GSE50081 dataset. (B) GSE30219 dataset. (C) GSE2total dataset.


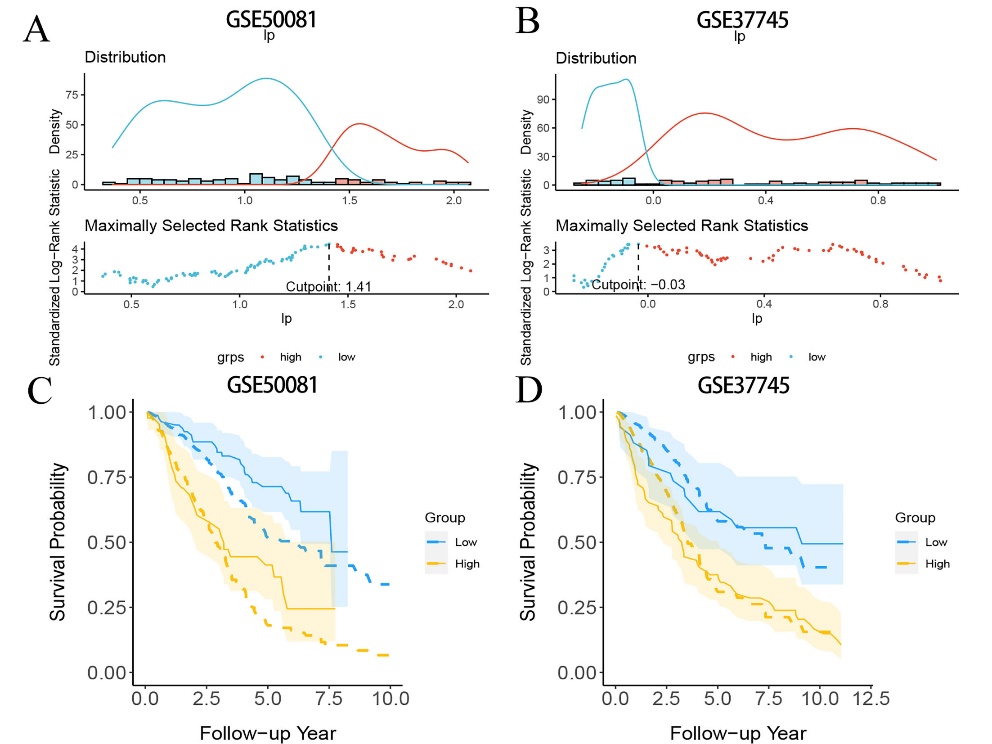


**Supplementary Figure 12** The calibration accuracy of comprehensive model in external validation data sets. The optimal cutoff value of PI: (A) GSE50081 dataset and (B) GSE37745 dataset. Predicted versus observed survival probability in two risk groups: (C) GSE50081 dataset and (D) GSE37745 dataset.


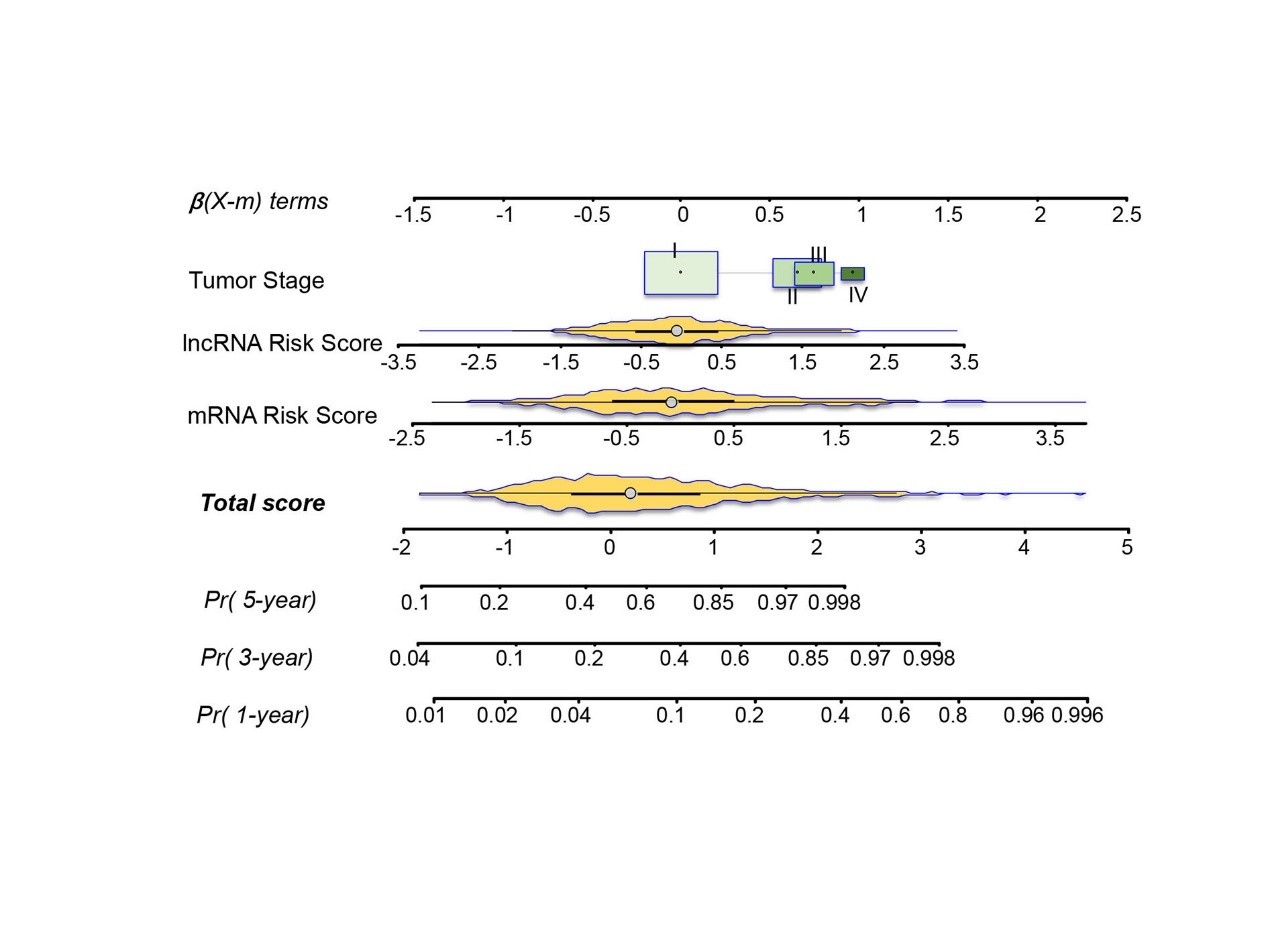


**Supplementary Figure 13** The nomogram of the comprehensive model predicts the probability of the 1-, 3-, and 5-year OS.


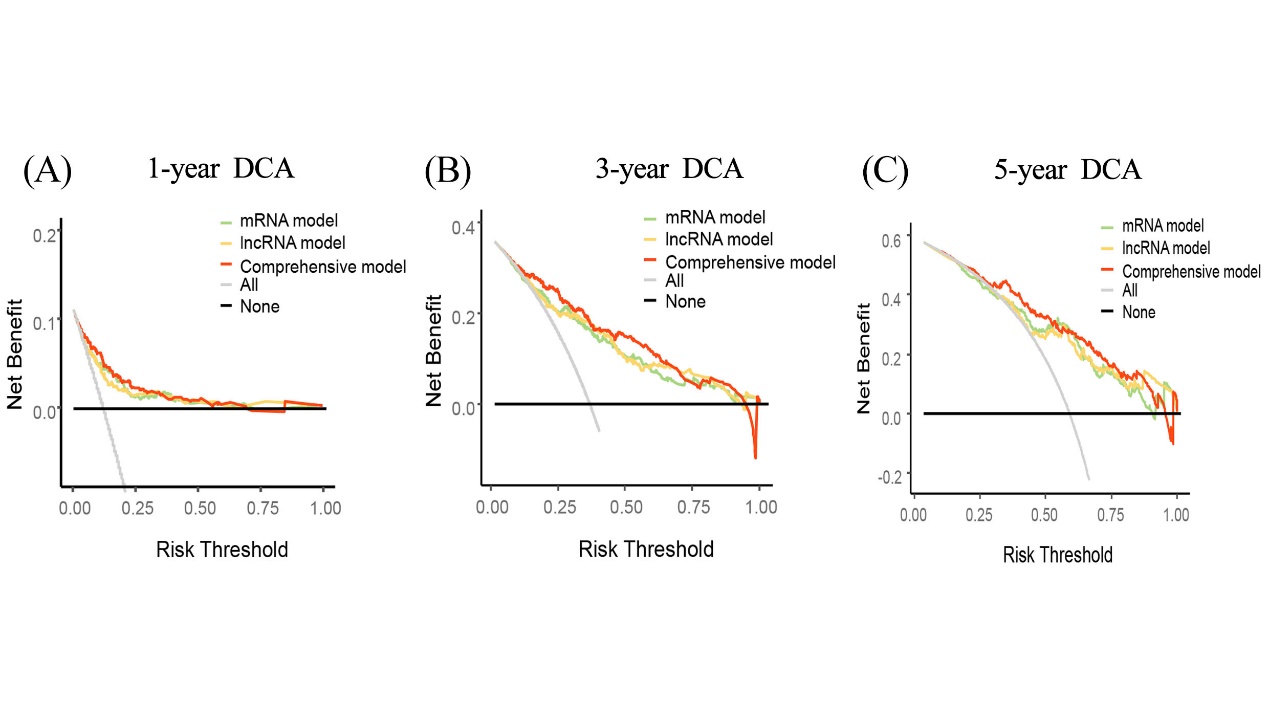


**Supplementary Figure 14** The DCA plots of three models in (A) 1-year, (B)3-year and (C) 5-year, respectively.


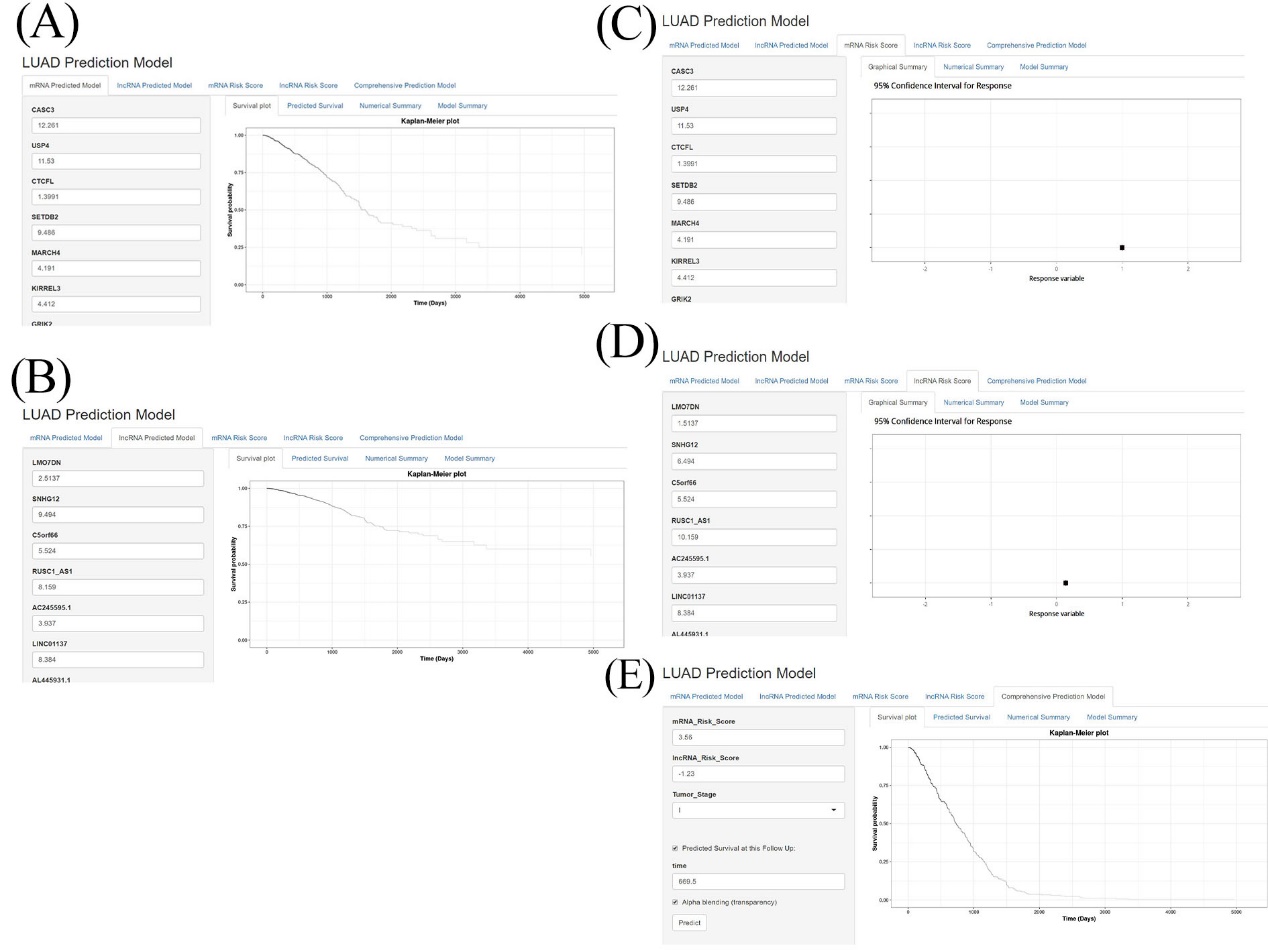


**Supplementary Figure 15** There are five calculators included in this website: (A)mRNA model, (B)lncRNA model, (C)mRNA Risk Score, (D)lncRNA Risk Score and (E)comprehensive prediction model.


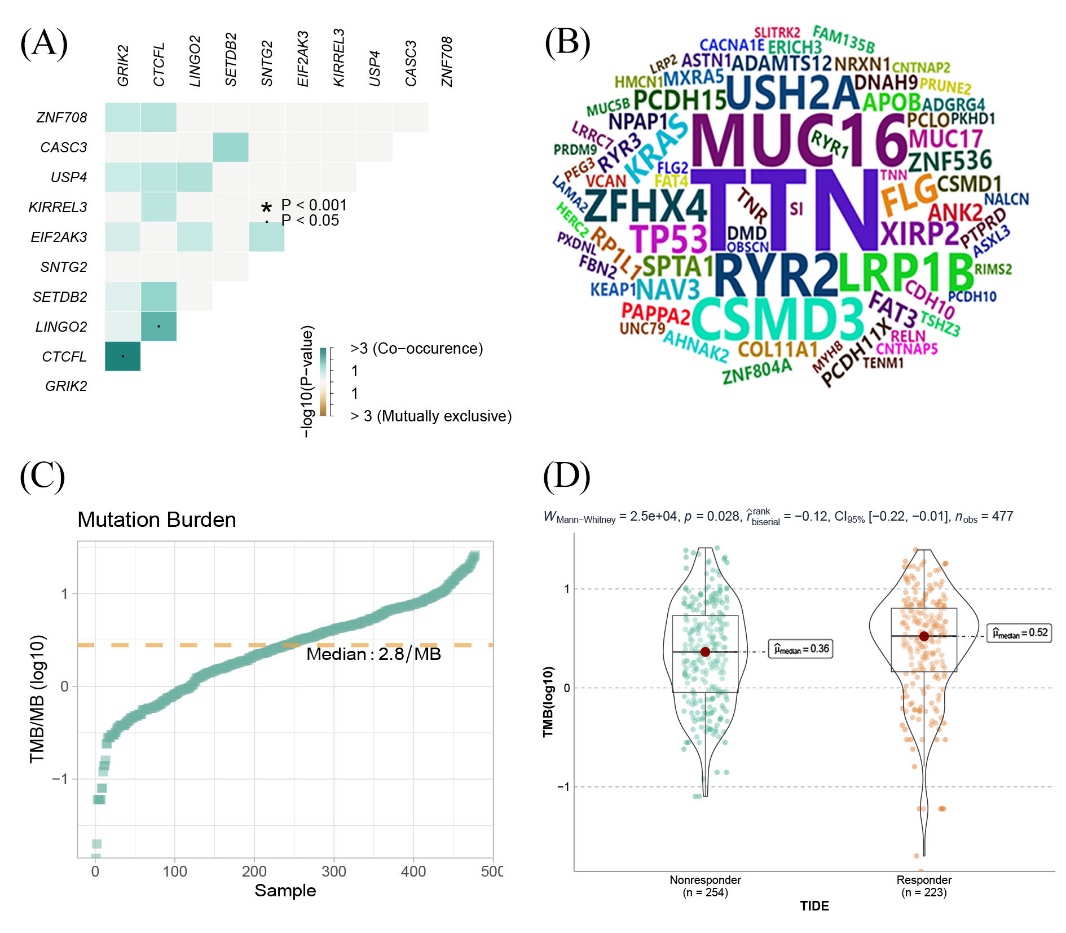


**Supplementary Figure 16.** Somatic mutations in lung adenocarcinoma patients in TCGA. (A) The heatmap of the mutually co-occurring and exclusive mutations of the 11 genes in mRNA model. (B) The gene word cloud of somatic mutation. (C) Total mutational burden (TMB) among tumor samples (n = 476). (D) TMB between TIDE predicted responders and non-responders.


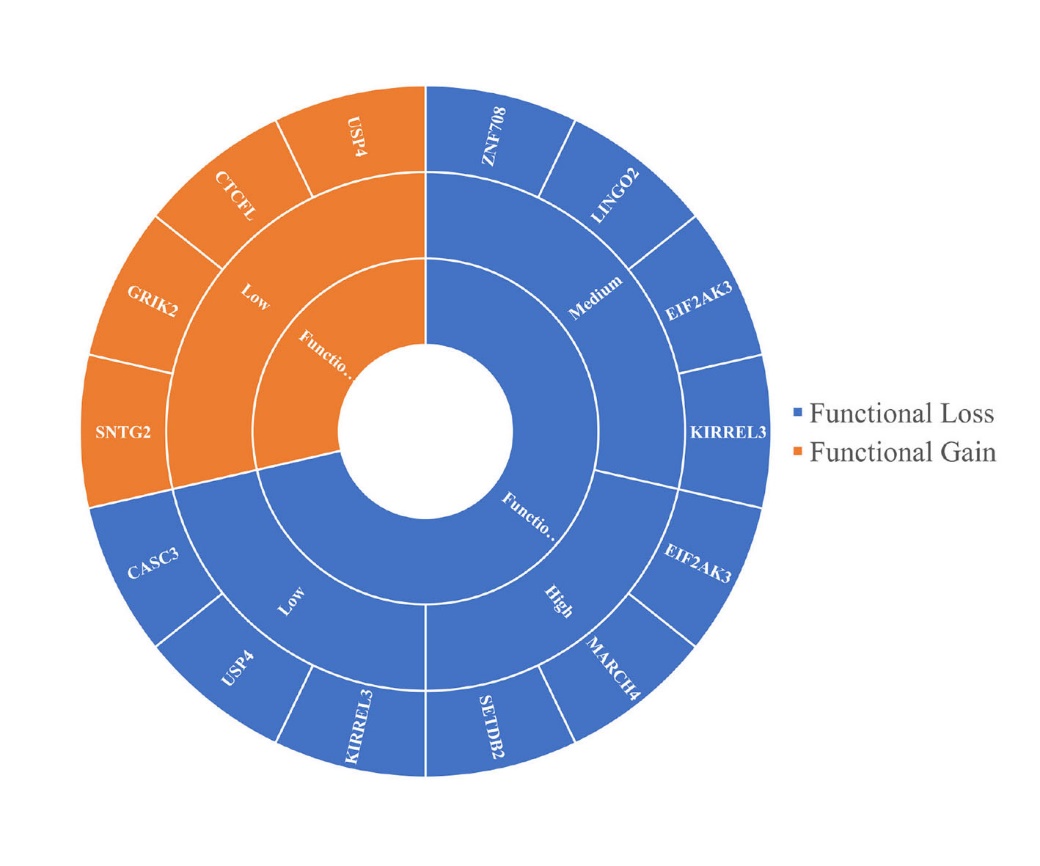


**Supplementary Figure 17** Visualization of mutation sites of 11 genes in m6AVar database.


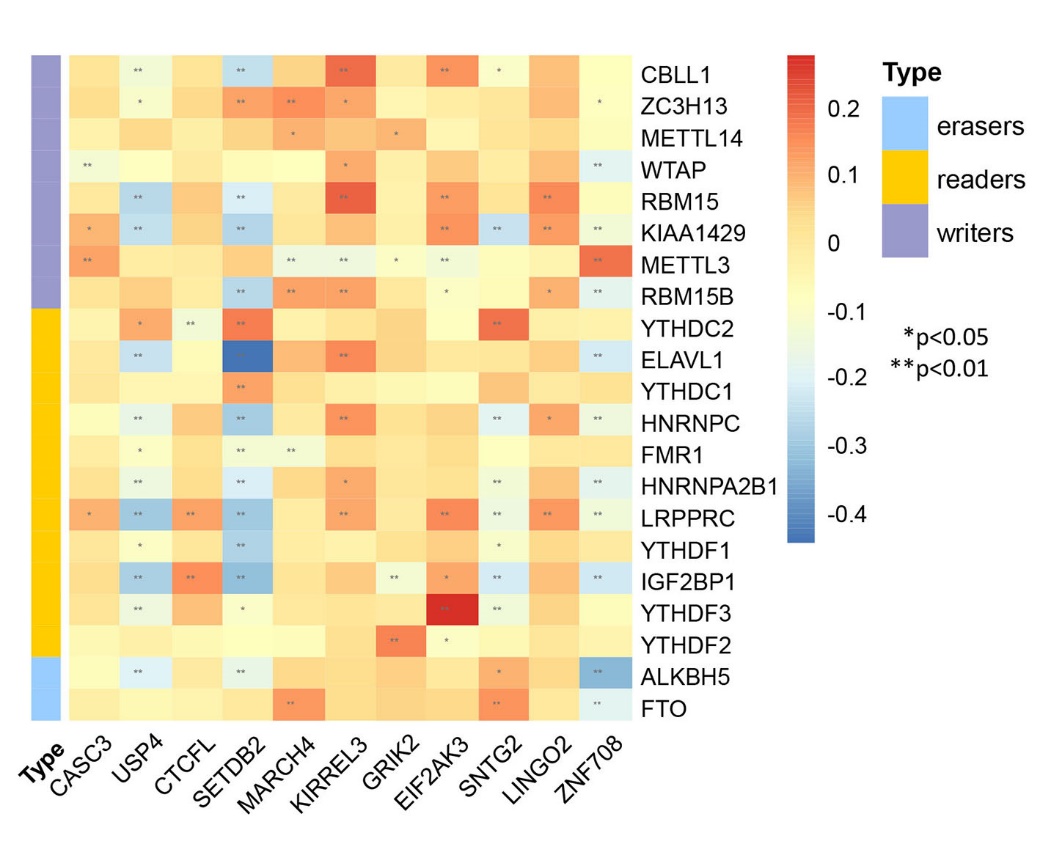


**Supplementary Figure 18** Heatmap of 11 mRNAs and 21 m6A regulatory factors.


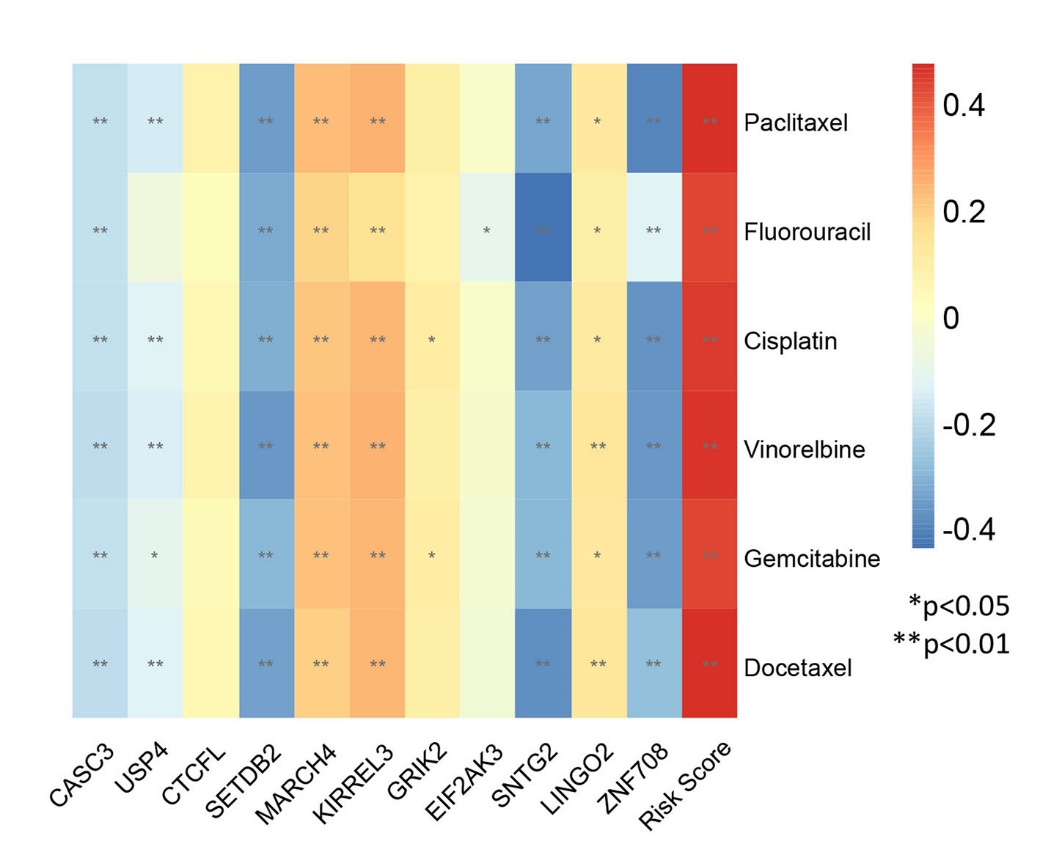


**Supplementary Figure 19** Heatmap of 11 mRNAs and risk score vs. 6 chemotherapeutic drugs.
